# Structural Impact of the Interaction of the Influenza A Virus Nucleoprotein with Genomic RNA Segments

**DOI:** 10.3390/v16030421

**Published:** 2024-03-09

**Authors:** Erwan Quignon, Damien Ferhadian, Antoine Hache, Valérie Vivet-Boudou, Catherine Isel, Anne Printz-Schweigert, Amélie Donchet, Thibaut Crépin, Roland Marquet

**Affiliations:** 1 Université de Strasbourg, CNRS, Architecture et Réactivité de l’ARN, UPR9002, 67000 Strasbourg, France; erwan.quignon@gmail.com (E.Q.); a.hache@ibmc-cnrs.unistra.fr (A.H.); v.vivet@ibmc-cnrs.unistra.fr (V.V.-B.); catherine.isel-griffiths@pasteur.fr (C.I.);; 2 Université Grenoble Alpes, CNRS, CEA, Institut de Biologie Structurale, 38000 Grenoble, Francethibaut.crepin@ibs.fr (T.C.)

**Keywords:** influenza A virus, NP, nucleoprotein, vRNA, RNA structure, RNA chaperon, chemical probing

## Abstract

Influenza A viruses (IAVs) possess a segmented genome consisting of eight viral RNAs (vRNAs) associated with multiple copies of viral nucleoprotein (NP) and a viral polymerase complex. Despite the crucial role of RNA structure in IAV replication, the impact of NP binding on vRNA structure is not well understood. In this study, we employed SHAPE chemical probing to compare the structure of NS and M vRNAs of WSN IAV in various states: before the addition of NP, in complex with NP, and after the removal of NP. Comparison of the RNA structures before the addition of NP and after its removal reveals that NP, while introducing limited changes, remodels local structures in both vRNAs and long-range interactions in the NS vRNA, suggesting a potentially biologically relevant RNA chaperone activity. In contrast, NP significantly alters the structure of vRNAs in vRNA/NP complexes, though incorporating experimental data into RNA secondary structure prediction proved challenging. Finally, our results suggest that NP not only binds single-stranded RNA but also helices with interruptions, such as bulges or small internal loops, with a preference for G-poor and C/U-rich regions.

## 1. Introduction

Influenza A viruses (IAVs) are respiratory viruses that constitute a major threat to animals and humans. In the human population, they cause seasonal flu epidemics that are responsible worldwide for 290,000 to 650,000 respiratory deaths yearly [1] and for occasional devasting pandemics. Like all members of the *Orthomyxoviridae* family, influenza viruses have a segmented negative strand RNA genome, which in the case of IAVs, consists of eight viral RNAs (vRNA) ranging from 890 to 2341 nucleotides (nts) in length. Each vRNA associates with multiple copies of the viral nucleoprotein (NP) and one copy of the viral polymerase, which consists of the PB2, PB1, and PA subunits, to form viral ribonucleoproteins (vRNPs) [2,3]. These vRNPs constitute the functional units for IAV transcription, replication, and packaging [2,4,5].

All IAV RNAs share a common organization, with a central coding region flanked by short segment-specific non-coding regions and terminal sequences named U12 and U13 (or Uni12 and Uni13) [2,6]. U12 and U13 are conserved amongst the eight vRNAs and amongst IAV isolates and constitute the promoter for the viral polymerase [2,6]. Stoichiometry analysis showed that vRNPs contain an average of one NP per 20 to 24 nts of viral RNA [7,8] while crosslinking and immunoprecipitation studies revealed that NP binding along vRNAs is neither uniform nor random, resulting in NP-rich and NP-poor vRNAs regions that differ between IAV isolates [9,10,11]. The monomeric 55 kDa NP protein can multimerize via a flexible tail loop and form loose polymers as well as rigid helices [12]. In vitro, the NP multimerization state is dependent on the salt concentration: at low salt concentration, NP is essentially monomeric, while it is mainly trimeric at 150 or 300 mM salt [13,14]. 

NP crystal structures revealed a putative RNA binding groove between the head and body domains of NP lined with conserved basic residues [12,15,16] required for RNA binding [15,17]. NP binds single-stranded RNA (ssRNA) with high affinity and little or no sequence specificity [14,18]. Each NP protomer binds ~12 nts [19], and IAV NP complexed with a 12-mer RNA assembles into long filaments resembling authentic vRNPs [14,20].

While the segmented nature of the IAV genome accelerates viral evolution, it complicates genome packaging, as fully infectious viral particles must contain one functional copy of each vRNA [2,4,5]. It is now generally accepted that IAVs selectively package one copy of each vRNA species [4,5,21]. The currently prevailing model is that the eight vRNPs are packaged as a supramolecular complex held together by direct base-pairing between the vRNA packaging signals that dictate selective packaging [5,21,22,23,24]. Initially, inter-vRNA interactions have been detected between ‘naked’ in vitro transcribed vRNAs [22,23,25], and one such interaction has been demonstrated to function in vRNA packaging [6,26]. More recently, genome-wide crosslinking studies identified numerous inter-vRNA interactions, mainly involving the internal parts of the vRNAs [27,28], but only one of these interactions was proven to be involved in packaging [27]. Thus, the current IAV packaging model is still far from being fully unveiled [21].

Additionally, there are indications that NP may impact vRNA packaging by altering the RNA structure and inter-vRNA interactions. Indeed, mutations in NP can induce segment-specific packaging defects, suggesting that NP might recognize features present in the vRNA packaging signals [29]. Early studies suggested that NP melts RNA secondary structures to expose the RNA bases [18,30], thus favoring inter-vRNA interaction, in agreement with a recent study showing that NP does not prevent inter-vRNA interactions [28]. Intramolecular secondary structures present in NP-poor regions also act as packaging signals [9]. Therefore, understanding how NP interacts with vRNA and affects its structure is a prerequisite for deciphering the IAV selective packaging mechanism. However, most studies analyzed these interactions using short-model RNAs [12,13,14,15], and detailed information on the structural impact of NP binding to vRNA is lacking. Many viral proteins affect RNA structure either transiently (i.e., as long as the protein is bound to the RNA) or permanently (i.e., the structure after removal of the protein differs from the structure of the RNA before the addition of the protein) [31,32,33]; the latter proteins are considered as RNA chaperones. Comparison of the structure of in vitro transcribed influenza vRNAs with deproteinated vRNPs using Selective 2′-Hydroxyl Acylation analyzed by Primer Extension (SHAPE) suggested that NP has limited permanent effects on RNA [27]. However, our knowledge of the RNA chaperone activity of NP remains limited.

Here, we used SHAPE to systematically compare the structure of the NS and M vRNAs of the WSN IAV before the addition of NP, in complex with NP, and after the removal of the protein. We observed that NP has limited but significant RNA chaperone activity. By contrast, NP dramatically affects the structure of the vRNAs to which it is bound. 

## 2. Materials and Methods

### 2.1. In Vitro Transcription of the M and NS vRNAs

For in vitro transcription of the M or NS vRNAs, pUC2000_WSN_M or pUC2000-WSN_NS plasmids, which contain the sequence coding for the M or NS segment of the A/WSN/1933 (H1N1) IAV strain under the control of a T7 RNA polymerase promoter, were linearized with Bsh1236I and Ech36II, respectively, and used as template. Twenty-five µg of linearized plasmids were incubated for 3 h at 37 °C in 300 µL of 40 mM Tris-HCl pH 8.0, 15 mM MgCl_2_, 50 mM NaCl, containing 4 mM of each NTP, 1 mM spermidine, 5 mM DTT, 1% triton X-100 (*v*/*v*), 160 U Rnasin (Promega, Charbonnière-les-Bains, France), 50 µg/mL BSA, and 0.5 µL recombinant pyrophosphatase (Roche, Mannheim Germany). Thirty-five µL of 10× DNase I buffer (Roche, Mannheim Germany), 3.5 µL of DNase I (Roche), and 11.5 µL of milliQ water were then added, and the solution was incubated further for 1 h at 37 °C. The reaction was then stopped by the addition of 150 µL of EDTA 250 mM, and a phenol/chloroform extraction was performed, followed by the addition of 3 vol. ethanol and 0.1 vol. sodium acetate 3 M at pH 5.0 and precipitation overnight at −20 °C. After centrifugation for 30 min at 4 °C at 21,000× *g*, the pellet was washed twice with cold (−20 °C) 70% ethanol, dried, and redissolved in 250 µL milliQ water and purified on a TSK Gel G4000SW (Tosh Bioscience GmbH, Griesheim, Germany) column at 1 mL/min in a buffer containing 0.2 M sodium acetate and 1% ethanol [22]. Fractions containing the RNA of interest were pooled, ethanol-precipitated, and redissolved in 100 µL milliQ water. Finally, NS and M vRNAs were quantified, and their integrity was checked by denaturing 8% polyacrylamide gel electrophoresis (PAGE).

### 2.2. Expression, Purification, and Characterization of the WSN NP Protein

Fifty µL of competent BL21 DE3 pLys cells were transformed with 50 ng of a pet22b plasmid coding for the WSN NP protein and one colony of transformed bacteria was used to seed 4 mL of LB medium supplemented with 4 µL ampicillin (100 mg/mL) and 4 µL chloramphenicol (30 mg/mL). The preculture was incubated overnight at 37 °C, at 160 rotations per minute (rpm). Two ml of this preculture were used to seed 200 mL of LB supplemented with 200 µL ampicillin and 200 µL chloramphenicol and were incubated overnight as above. Finally, this culture was used two seed two two-liter cultures at an optical density (OD) of ~0.05, which were further incubated as above. At an OD of 0.6 to 0.8, the cultures were cooled in a water/ice bath until they reached 18 °C. The cultures were kept at 4 °C for 1 h, then 30 min at 18 °C, and induction was performed overnight with 0.3 mM IPTG at 18 °C. Cells were centrifuged for 15 min at 4000× *g* and immediately used for protein purification or frozen at −20 °C.

Bacterial pellets were resuspended in 80 mL lysis buffer (50 mM Tris-HCl pH 7.5, 300 mM NaCl, 1M NDSB, 2 mM β-mercaptoethanol) and bacteria were lysed by sonication with a VC-500 sonicator (Sonics & Materials, Fisherscientific, Illkirch, France, 500 W, 20 kHz) and a V1A probe (Sonics & Materials, 1 s on, 1 s off, 70% charge, 3 × 5 min)). Lysates were centrifuged for 1 h at 7 °C at 11,000× *g,* and the supernatants were pooled together and adjusted to 20 mM imidazole BioUltra (Sigma, Saint-Quentin-Fallavier, France). A HisTrap FF crude 5 mL column (GE Healthcare Bio Science, Uppsala, Sweden) attached to a Biologic DuoFlow station (Biorad, Marnes-la-Coquette, France) was equilibrated with a low salt buffer (50 mM Tris-HCl pH 7.5, 300 mM NaCl, 20 mM imidazole BioUltra, 2 mM β-mercaptoethanol) and the lysate was loaded. Unless specifically indicated, all chromatographic steps were performed at a flow rate of 2 mL/min. The column was first washed with 20 mL 50 mM Tris-HCl pH 7.5, 1 M NaCl, 20 mM imidazole BioUltra, 2 mM β-mercaptoethanol, then with 50 mL of low salt buffer, and the protein was eluted with 15 mL 50 mM Tris-HCl pH 7.5, 300 mM NaCl, 800 mM imidazole BioUltra, 2 mM β-mercaptoethanol. 

The fractions containing the NP protein were pooled and loaded on a 5 mL HiTrap Heparin column (GE Healthcare) equilibrated with buffer A (50 mM Tris-HCl pH 7.5, 300 mM NaCl, 2 mM β-mercaptoethanol). After loading the sample, the column was washed with 20 mL buffer A, and then the proteins were eluted in 30 mL of a linear gradient going from buffer A to buffer B (50 mM Tris-HCl pH 7.5, 2 M NaCl, 2 mM β-mercaptoethanol). The most concentrated fractions were pooled (with a maximum of 15 mL) and dialyzed overnight against the gel filtration buffer (20 mM Tris-HCl pH 7.5, 150 mM NaCl, 5 mM β-mercaptoethanol).

Next, the samples were concentrated to ~1.2 mL on an Amicon Ultra-15 device (Millipore, Molsheim, France, 30 kD cut-off) and divided into three parts that were sequentially loaded on a Superdex 200 Increase 10/300 GL column (GE Healthcare) equilibrated with the gel filtration buffer and elution was performed with 30 mL of the same buffer at 0.5 mL/min. The fractions of interest were selected and analyzed on a 12% SDS PAGE. The fractions containing pure NP were pooled and dialyzed in a Slide-A-LyzerTM G2 cassette (Thermofisher Scientific, Illkirch, France, 10 kD cut-off, 15 mL) against 50 mM HEPES pH 8.0, 50 mM KCl, 5 mM β-mercaptoethanol. Finally, the protein solution was centrifuged at 21,000× *g* for 10 min at 7 °C, and the top half of the solution was recovered in a new tube, while the lower part of the solution, possibly containing protein aggregates, was discarded. 

Prior to the in vitro assays, purified NP was characterized by DLS using a DynaPro Nanostar (100 mW He-Ne laser; Wyatt Technologies, Toulouse, France) in a 1-µL quartz cuvette (WNQC01-00, Wyatt Technologies) at 20 °C as previously described [34]. By assimilating the protein in solution to spheres, the diffusion coefficient (D) was correlated to the hydrodynamic radius (Rh) of the molecules in solution using the Stokes–Einstein equation.

For band-shift experiments, 10 pmol NS or M vRNA were denatured (2 min at 90 °C, followed by 2 min on ice) and refolded 30 min at 37 °C in 200 mM Bicine pH 8.3 (at 25 °C), 150 mM KCl, and 5 mM MgCl_2_. The refolded vRNA was incubated for 30 min at 37 °C with NP protein at ratios ranging from 1 NP per 100 nts to 1 NP per 15 nts. The samples were then loaded on a 1% agarose gel containing 312.5 ng/mL ethidium bromide and migrated for 45 min at 100 V at 4 °C in 45 mM Tris-base, 45 mM boric acid, 0.1 mM MgCl_2_. 

### 2.3. Modification of the M and NS vRNAs 

In vitro transcribed M and NS vRNAs were modified with N-methylisatoic anhydride (NMIA) under three conditions: (i) in vitro transcribed RNA in the absence of NP (NoNP), (ii) RNA in complex with NP (Comp), and (iii) RNA incubated with NP, which was subsequently removed using proteinase K treatment before RNA modification (ProtK). For each condition, a negative control with DMSO was conducted in parallel with the modification reaction performed from a stock solution of 57.75 mM NMIA in anhydrous DMSO. For the NoNP condition, 8 or 10 pmoles of NS and M vRNA, respectively, were folded in 50 mM HEPES pH 8.0, 150 mM KCl, 5 mM MgCl_2_ for 30 min at 37 °C before RNA modification for 50 min at room temperature with 1.65 mM NMIA. After twofold dilution with water and the addition of 1 µg glycogen, the samples were ethanol-precipitated for 30 min at −80 °C and centrifuged 30 min at 7 °C at 21,000× *g*; the pellets were washed twice with 300 µL cold 80% ethanol, dried and redissolved in 28 (NS vRNA) or 35 (M vRNA) µL milliQ water. 

For the Comp and ProtK conditions, KCl and MgCl_2_ were added to the NP solution in order to reach final concentrations of 150 mM and 5 mM, respectively, and the vRNA/NP complexes were formed at 37 °C for 30 min at a molar ratio of 1 NP protein for 20 nts. For the Comp condition, RNAs were modified as described for the NoNP condition, and samples were further incubated with 1 µL of proteinase K at 18 mg/mL (Roche) per 15 µL of solution for 1 h at 37 °C in the presence of 10 mM ribo-vanadyl complexes (Sigma-Aldrich). The samples were then extracted three times with phenol/chloroform (*v*/*v*) and ethanol-precipitated. For the ProtK condition, the samples were first treated with proteinase K, phenol/chloroform-extracted, and ethanol-precipitated as described above for the Comp condition, then modified as described for the NoNP condition.

### 2.4. Reverse Transcription and cDNA Analysis 

For each vRNA, the same amount of NMIA-modified RNA and negative control RNA were split into 3 (NS vRNA) or 4 (M vRNA) pairs of tubes. After incubation for 30 min at 50 °C with Vic 5′-labeled primers NS30V, NS341V or NS621V for the NS vRNA (Table 1) or M20V, M312V, M622V or M843V for the M vRNA (Table 1), reverse transcription was performed in a 20 µL volume containing 1× Superscript II buffer, 10 mM DDT, 0.75 mM dNTP and 40 U Superscript II (Thermofisher Scientific). In parallel, sequencing reactions were performed on unmodified vRNA using Ned 5′-labeled primers (Table 1) in 20 µL containing AMV RT buffer 1×, 2 pmoles RNA, 2.5 µM ddGTP, 5 µL G10 (1 mM dATP, 1 mM dCTP, 1 mM TTP, 0.25 mM dGTP) and 4 U AMV RT (Promega). After phenol/chloroform extraction (*v*/*v*), the sequencing reactions were added to the tubes containing the NMIA-modified and the negative control RNAs. The samples were extracted with chloroform (*v*/*v*), ethanol-precipitated, and redissolved in 10 µL HiDi Formamide (Applied Biosystems, Termofisher Scientific, Illkirch, France). The samples were heated for 5 min at 90 °C, vortexed, heated for 2 min at 90 °C, kept on ice for 5 min, centrifuged for 5 min at 6000× *g*, loaded on a 96 well plate, and analyzed on a 3130xl Genetic Analyzer (Applied Biosystems) capillary sequencer.

### 2.5. Data Analysis

Following capillary electrophoresis, the electropherograms were analyzed with the QuShape v1.0 software in order to obtain reactivity profiles for vRNAs M and N under the NoProt, Comp and ProtK conditions [35]. SHAPE reactivity values were used as constraints to model the vRNA secondary structure using RNAStructure (version 6.4) [36] with the default values of −0.6 kcal/mol and 1.8 kcal/mol [37] for intercept and slope, respectively, and secondary structure were using VARNA (version 3.93) [38]. Secondary structures predicted with RNAStructure v6.4 were compared using RNAStructViz v2.14.18 [39].

## 3. Results

### 3.1. Characterization of the WSN NP Protein

Ionic strength is known to promote oligomerization of the IAV NP protein [13,16,40]. Here, we purified and stored NP at a low salt concentration (see Section 2).

**Figure 1:**
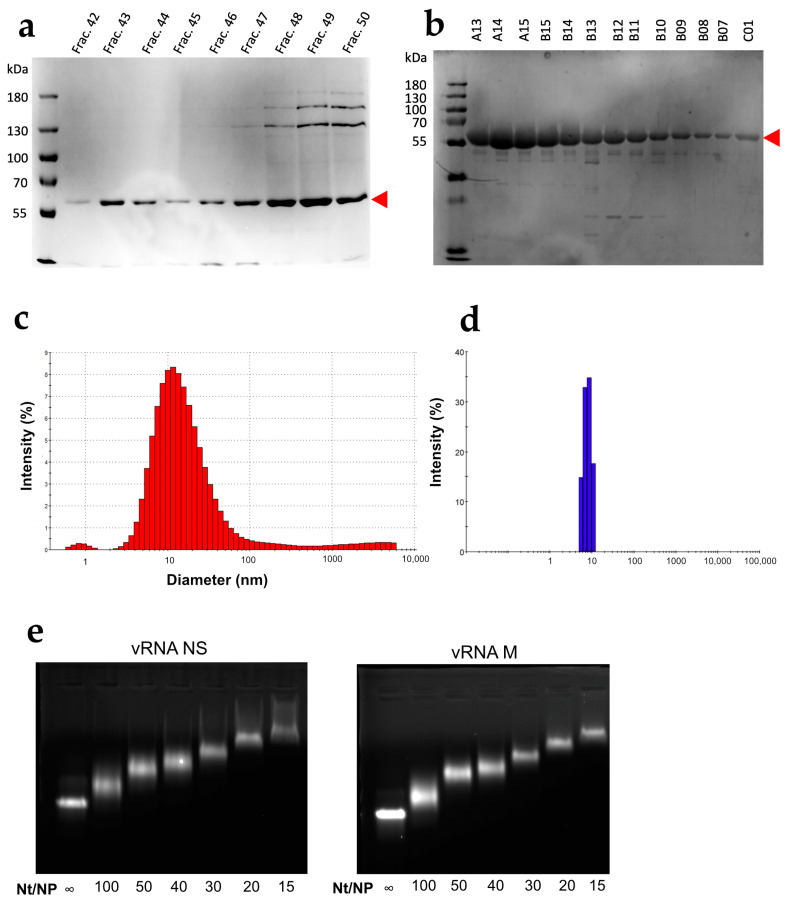
Characterization of the WSN NP protein. Fractions obtained after the last chromatographic step performed at high [40] (**a**) or low ionic strength (**b**) were analyzed using SDS PAGE. The DLS profiles of the proteins purified in (**a**,**b**) are shown in panels (**c**,**d**), respectively. (**e**) Ten pmoles of NS (**left**) or M (**right**) vRNAs were incubated with increasing amounts of NP protein purified at low ionic strength at the nt/NP ratios indicated below the lanes and analyzed by electrophoresis through a 1% agarose gel containing 0.5 µg ethidium bromide per ml.

Compared to previous purification protocols using high ionic strength [40], the protocol used here increased the final NP yield by about 9-fold. Compared to the protein purified at high ionic strength (Figure 1a), the NP recovered after 3 chromatographic steps using the present protocol was purer (>95% pure) (Figure 1b). In addition, the DLS profile of the protein purified at low ionic strength exhibits a single narrow peak corresponding to a monomer (Figure 1d) that contrasts with the broad peak observed with the protein purified at high ionic strength (Figure 1c). Furthermore, the addition of increasing amounts of NP to either the NS or M vRNA produced a gradual shift of the RNA during native electrophoresis through agarose gels, and discrete bands were observed at all nt/NP ratios (Figure 1e). This result indicated that the purified NP is functional and binds vRNAs in a non-cooperative manner to form homogenous complexes.

### 3.2. NP Has Limited but Significant RNA Chaperone Activity

Not all RNA molecules spontaneously adopt their functional conformation in cells and viral particles, and some of them require the assistance of proteins to do so. By analogy with protein chaperones, RNA chaperones were initially defined as proteins that bind transiently and non-specifically to RNA and resolve kinetically trapped, misfolded conformers [41]. However, any protein that shows activity in any of the assays designed to test RNA chaperone activity [41] is usually considered an RNA chaperone even though its binding to RNA is not transient. Over time, numerous structural [31,33,42,43] and non-structural [32,44] viral proteins have been considered RNA chaperones, even though most of them do not spontaneously dissociate from RNA. The effect of such RNA chaperones can be assessed by comparing the RNA structure before the addition of the protein and after the removal of the protein by SDS or/and proteinase K treatment [27,32,42].

We thus analyzed the structure of the NS and M vRNAs before the addition of NP (No-NP condition, see Section 2) and after the removal of the protein by proteinase K treatment (ProtK condition) by SHAPE using NMIA [45,46]. NMIA modifies the ribose of flexible unpaired nts [45,46,47], and SHAPE reactivities can be implemented as pseudo-energies to improve RNA secondary structure predictions [48,49]. Mean SHAPE reactivity values of the NS and M vRNAs under the NoNP and ProtK conditions were obtained from highly correlated triplicate experiments (median = 0.89, range = 0.67–0.97) (Data S1 and Table S1).

SHAPE reactivity values of RNA were used to define three broad categories: nts with low reactivity (<0.4), usually involved in RNA secondary or tertiary structures (or protected by proteins in RNA/protein complexes); highly reactive nts (>0.8), usually unpaired and thus present in apical loops, internal loops, bulges or single-stranded junctions; nts with intermediate reactivity values that can be part of unstable secondary structures or structured loops [36,48,49,50].

We thus first compared the reactivity of NS and M vRNAs under the NoNP and ProtK conditions (Table 2).

Table 2 shows that 79.7 and 75.4% of nts of the NS and M vRNAs, respectively, belonged to the same reactivity category under the NoNP and ProtK conditions; the reactivity of 18.0 and 22.8% of nts of the NS and M vRNAs, respectively, differed by one category (from intermediate to low or intermediate to high or vice-versa), and the reactivity of 2.3 and 1.8% of nts of the NS and M vRNAs, respectively, went from low to high or vice-versa. Notably, 9 and 37 nts of the NS vRNA and 31 and 50 nts of the M vRNA were highly reactive uniquely under the NoNP or ProtK condition, respectively (Table 2).

This analysis suggests that while the NS and M vRNAs both adopt similar structures under the NoNP and ProtK conditions, these structures differ to some degree. To test this hypothesis, we used the SHAPE reactivity data as constraints to model the secondary structures of these vRNAs using RNAStructure [49], revealing that most local secondary structure elements are common to the two structures (Figure S1a,b). Indeed, both structures share 13 stems capped by an apical loop, while two are unique to the NoNP structure, and one only exists solely in the ProtK structure (compare Figure S1a,b).

**Figure 2:**
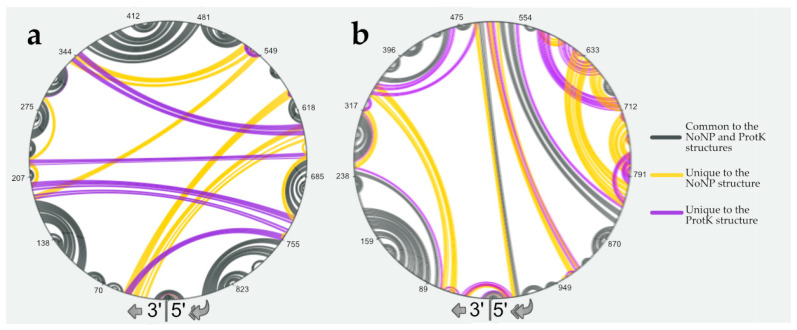
Comparison of the predicted secondary structures under the NoNP and ProtK conditions for the NS (**a**) and M (**b**) vRNAs. Structural elements that are common to the NoNP and ProtK conditions are indicated in grey, and those unique to the NoNP and ProtK conditions are in yellow and purple, respectively. Nucleotides of the vRNA are numbered from 3′ to 5′ as per convention in the field of negative strand viruses.

In order to confirm this, we compared the predicted NoNP and ProtK secondary structures of vRNA NS using RNAStructViz [39] (Figure 2). This analysis indicated that 550 and 530 nts out of the 890 nts of the NS vRNA are base-paired under the NoNP and ProtK conditions, respectively. Of these, 436 form identical base pairs under both conditions. Remarkably, almost all short-range interactions are maintained in the NoNP and ProtK conditions (Figure 2a). By contrast, the predicted long-range interactions that maintain the overall secondary structure of the NS vRNA drastically differ in the NoNP and ProtK conditions (Figure 2a).

We performed a similar analysis on the M vRNA. When we used the SHAPE data as constraints to model the secondary structure of the M vRNA under the NoNP and ProtK conditions, we observed that the predicted structures are more different than in the case of the NS vRNA (Figure S2). The two M vRNA structures share 13 stems capped by an apical loop in common, but three are unique to the NoNP structure, and seven only exist in the ProtK structure (Figure S2). Comparison of the NoNP and ProtK structures of the M vRNA using RNAStructViz [39] indicated that 618 and 604 nts out of the 1027 nts of the M vRNA are base-paired under the NoNP and ProtK conditions, respectively. Of these, 378 form identical base pairs under both conditions. These results indicated that the NP induced more permanent structural rearrangements in the M vRNA than in the NS vRNA (Figure 2). Moreover, unlike in the NS vRNA, NP induced mainly short-range and intermediate-range structural rearrangements in the M vRNA, while the long-range interactions were less affected (Figure 2b).

**Figure 3:**
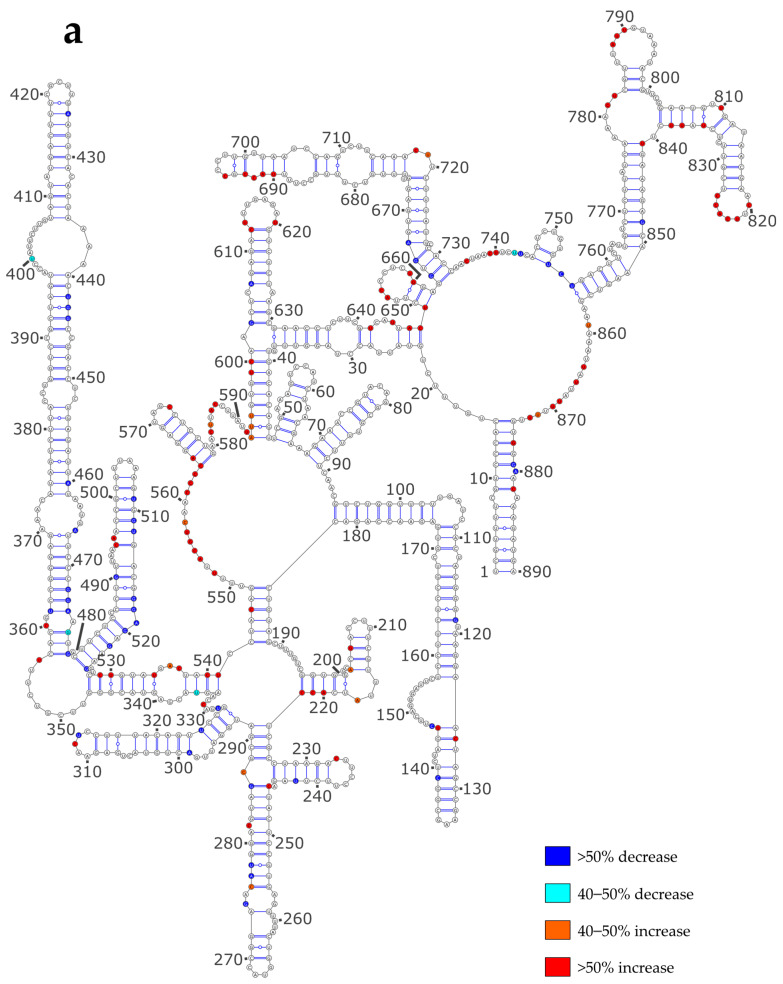

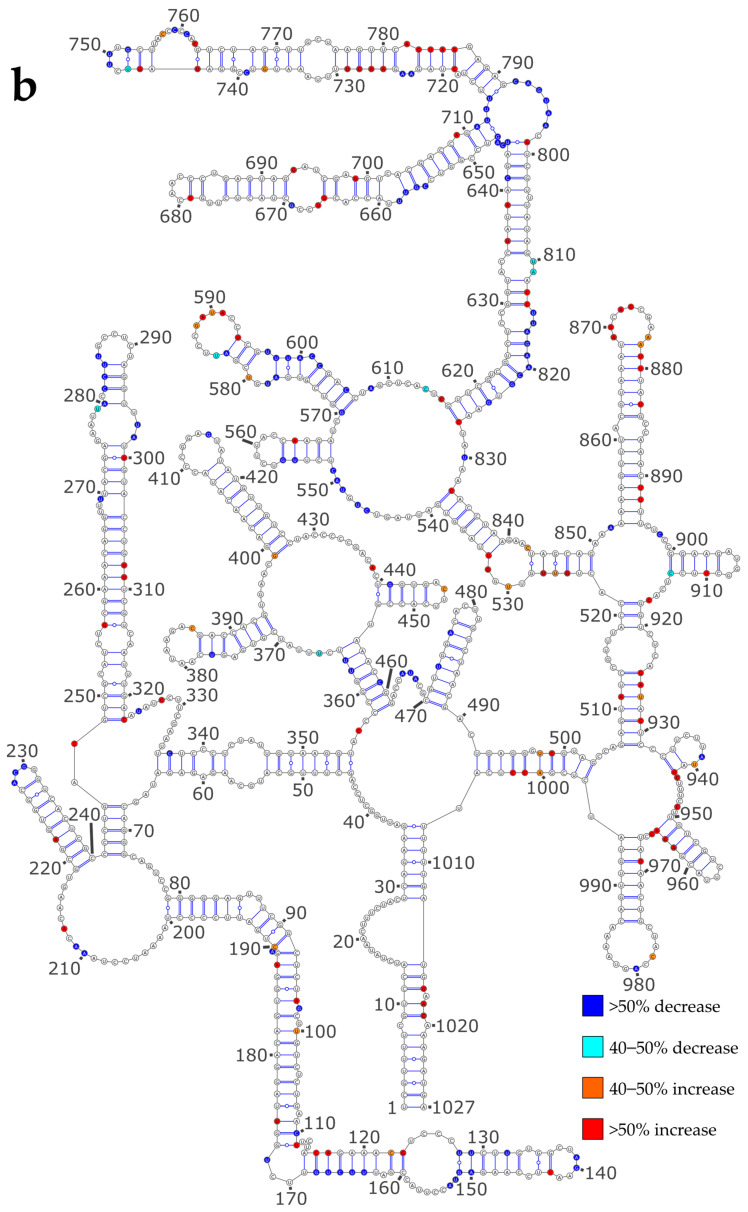
SHAPE reactivity differences under the ProtK versus NoNP condition. (**a**) NS vRNA; (**b**) M vRNA. Nucleotides of the vRNA are numbered from 3′ to 5′ as per convention in the field of negative strand viruses.

Next, we checked whether the differences in the proposed secondary structure models between the NoNP and ProtK conditions correlate with local SHAPE reactivity differences. To that aim, we reported the most pronounced reactivity changes on the secondary structures predicted under the NoNP condition for the NS and M vRNAs (Figure 3a and Figure 3b, respectively).

In the case of the NS vRNA, most increased reactivities in the ProtK condition compared to the NoNP condition were observed in single-stranded nts (e.g., in the 549–563, 580–592, 718–720, 733–745, 778–782, and 857–874 internal loops and the 613–620, 652–659, 786–796, 819–826 apical loops), whereas diminished reactivities were mainly observed in stems (e.g., in stems 250–255/276–281, 362–369/467–474, 480–485/520–525, 487–491/514–518, 497–502/507–513, and 663–666/729–732) (Figure 3a). These differences do not support the idea that the NS vRNA adopts different structures under the two conditions but suggest that most local structures are the same under both conditions (although the 3D structures might be different). However, a few nts that form stems in the NoNP secondary structure had a higher reactivity under the ProtK condition, namely the 23–25/646–648, 39–47/593–601, 196–199/218–222, 333–336/538–541, 341–347/528–534, 689–691/702–704 and 804–808/835–839 stems (Figure 3a), suggesting that these stems do not exist under the latter condition.

Indeed, the first five of these helices, which correspond to long-range interactions (except for the 196–199/218–222), were not predicted when the ProtK SHAPE data were used as constraints for secondary structure modeling (Figure S1 and Figure 2a). Despite their high relative SHAPE reactivity under the ProtK condition compared to NoNP, the two short-range stems 689–691/702–704 and 804–808/835–839 were predicted to exist under both conditions (Figure S1 and Figure 2a). Nevertheless, the ProtK model of vRNA NS does not contradict the experimental data, as the absolute SHAPE reactivities of these regions remained low and consistent with base-pairing under the ProtK conditions (Figure S1b).

Hence, our SHAPE data show that while most secondary structure elements of the NS vRNA exist under both the NoNP and ProtK conditions, a few long-range interactions differ between these two conditions, indicating that NP has a limited but real RNA chaperone activity that could have a major biological impact as long-range interactions play a crucial role in the three-dimensional RNA structure.

Similarly, we observed that under the ProtK conditions some of the enhanced reactivities in the M vRNA conditions were located in unpaired regions (e.g., 529–532, 584–593, 869–877), whereas some diminished reactivities were observed in stems (e.g., in stems 128–133/146–151, 280–284/292–295, 358–364/455–461, 470–477/482–489, 570–577/598–606, and 617–627/817–827) (Figure 3b). However, compared to the NS vRNA, a greater proportion of increased reactivities in the M vRNA under the ProtK condition were observed in stems of the NoNP secondary structure (compare Figure 3b with Figure 3a). Moreover, in the M vRNA, several diminished reactivities under the ProtK conditions were located in regions that are unpaired in the NoNP secondary structure model (Figure 3b). Thus, a direct comparison of the SHAPE data suggested that the NP protein has a more pronounced RNA chaperone activity on the M vRNA than on the NS vRNA (Figure 3), consistent with the comparison of the secondary structure models predicted using the SHAPE data as constraints (Figure S2 and Figure 2). A comparison of the predicted secondary structure models of the M vRNA M the NoNP and ProtK conditions shows that three regions are largely remodeled under the ProtK conditions: (1) the region corresponding to stems 492–496/1001–1005, 46–53/347–354, 58–64/336–342, and 248–254/316–322 and the single stranded junctions between these helices, (2) the region between nts 568 and 832, and (3) the region encompassing nts 917–942 and 507–521 (Figure S2 and Figure 2). In each of these regions, at least one helix is destabilized under the ProtK condition compared to NoNP (Figure 3b), and the secondary structure models of the M vRNA exhibit no local disagreement with the SHAPE reactivity data (Figure S2), supporting the validity of our conclusions.

Hence, our data indicate that the NP RNA chaperone activity is somewhat more pronounced on the M vRNA compared to the NS vRNA, although the structural rearrangements in the former RNA are more localized.

### 3.3. NP Has a Major Impact on the RNA Structures to Which It Binds

Next, we used NMIA to study the RNA chaperone-independent structural impact of NP on the vRNA structure. To that aim, we probed the vRNA/NP complexes (Comp condition) and compared the SHAPE values obtained under this condition with those obtained under the NoNP and ProtK conditions. The Comp SHAPE profiles of the NS (Figure S3a) and M (Figure S3b) vRNAs markedly differ from the NoNP and ProtK profiles, suggesting that NP substantially modifies the vRNA structure. This result was confirmed by comparing the classified (low, intermediate, or high) SHAPE values of the NS and M vRNAs between the NoNP and Comp (Table 3) or ProtK and Comp (Table 4) conditions.

Only 54.9 (M vRNA) to 58.3% (NS vRNA) of nts belonged to the same reactivity category under the NoNP and Comp conditions; the reactivity of 30.0 (NS vRNA) to 34.8% (M vRNA) of nucleotides differed by one category (from intermediate to low or intermediate to high or vice-versa), and the reactivity of 10.3 (M vRNA) to 11.9% (NS vRNA) of nts went from low to high or vice-versa. Notably, 67 and 58 nts of the NS vRNA and 77 and 64 nts of the M vRNA were highly reactive uniquely under the NoNP or Comp condition, respectively (Table 3). Thus, the SHAPE reactivity values of the NS and M vRNAs are much more different between the NoNP and Comp conditions (Table 3) than between the NoNP and ProtK conditions (Table 2). Of note, the SHAPE reactivity differences between the ProtK and Comp conditions (Table 4) are very similar to those observed between the NoNP and Comp conditions (Table 3): 52.1 (M vRNA) to 57.1% (NS vRNA) of nts fell into the same reactivity category under the ProtK and Comp conditions, 29.5 (NS vRNA) to 35.3% (M vRNA) of nts differed by one category), and 12.6 (M vRNA) to 13.4% (NS vRNA) of nts differed by two categories (from low to high or vice-versa) (Table 4). Altogether, our SHAPE data indicate that NP has a major structural impact when it binds to the NS or M vRNA; most of this effect is lost when NP is removed by ProtK treatment.

There is no medium or high-resolution structure of the vRNA/NP interactions since, for instance, vRNA is not visible in the cryo EM structures of authentic vRNPs [3,51,52]. However, NP crystal structures revealed a putative RNA binding groove between the head and body domains of NP lined with conserved basic residues that most likely interact with the ribose phosphate moieties of RNA [12,15,16,17]. Furthermore, a recent sub-nanometric structure of a model helical IAV nucleocapsid identified RNA densities adjacent and between the positively charged NP surfaces [20]. Therefore, since NMIA, as all SHAPE reagents, modifies the 2′OH of unpaired and flexible nucleotides, it is expected that the binding of NP decreases the SHAPE reactivity of the nts interacting with the protein. Thus, unreactive nts can be either base-paired or interact with NP, while highly reactive nts are neither base-paired nor interact with NP.

**Figure 4:**
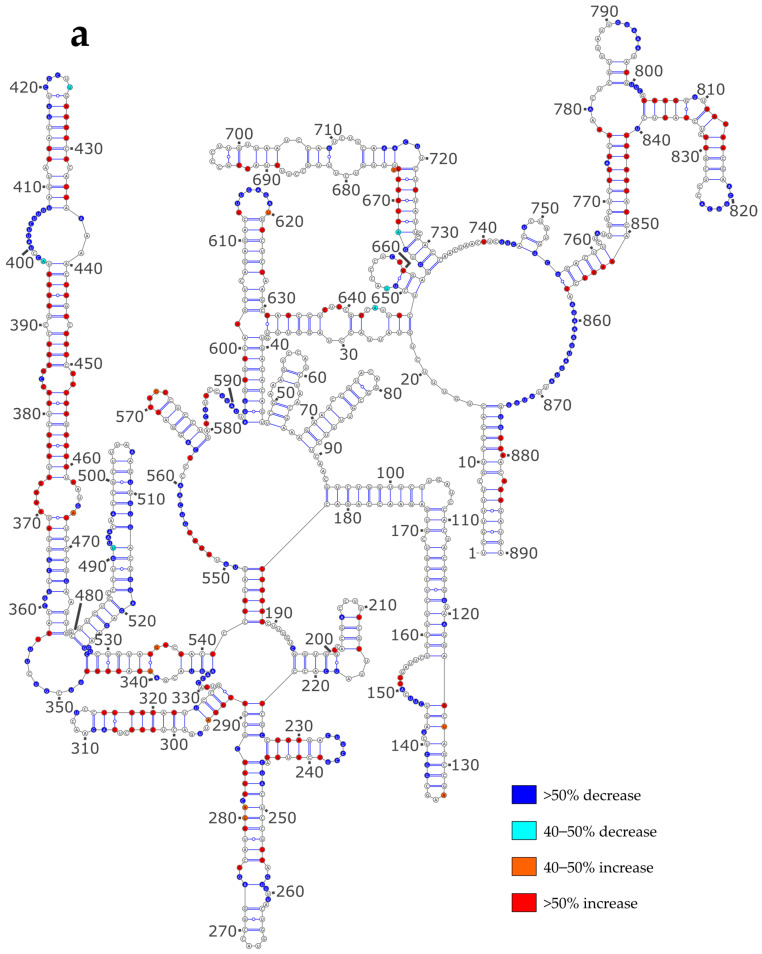

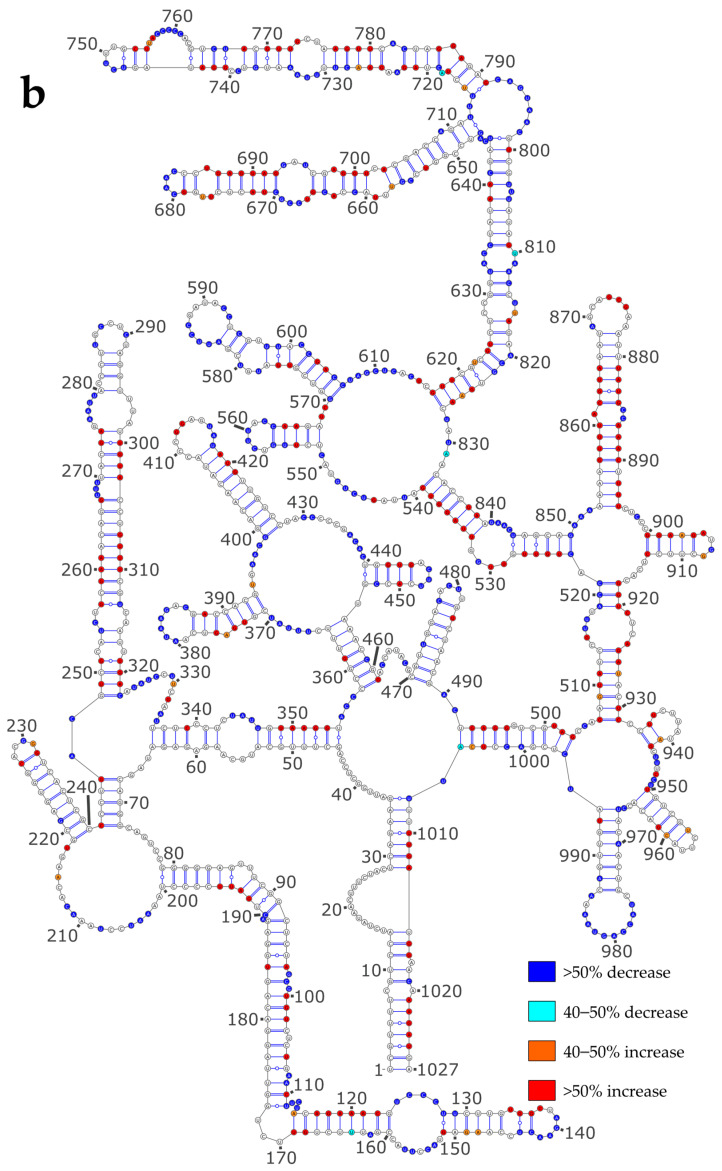
SHAPE reactivity differences under the Comp versus NoNP condition. (**a**) NS vRNA; (**b**) M vRNA. Nucleotides of the vRNA are numbered from 3′ to 5′ as per convention in the field of negative strand viruses.

In order to assess the impact of NP on the vRNA structure, we reported the differences in reactivity of the NS and M vRNAs between the NoNP and Comp conditions on the secondary structure models obtained using the NoNP SHAPE data as constraints (Figure 4). When comparing the NoNP and Comp reactivity values, it appears that, both for NS (Figure 4a) and M (Figure 4b) vRNAs, the addition of NP mostly induces SHAPE reactivity decreases in single-stranded regions, and reactivity increases in helices. In some cases, the protections induced by NP in single-stranded regions extend by one or a few base-pairs in the adjacent stem: e.g., nts 331-336 and 417-420 in the NS vRNA (Figure 4a) and 124–129, 139–144, 344–347, 416–418, 555–563, 583–595, 605–612, 632–635, 667–670, 728–734, for the M vRNA (Figure 4b). Furthermore, the stretches of contiguous nucleotides showing an increase in SHAPE reactivity upon NP binding are usually located in close vicinity to protected regions (Figure 4). This pattern of reactivity change suggests that NP usually binds to single-stranded regions of vRNAs and destabilizes the adjacent stems.

**Figure 5:**
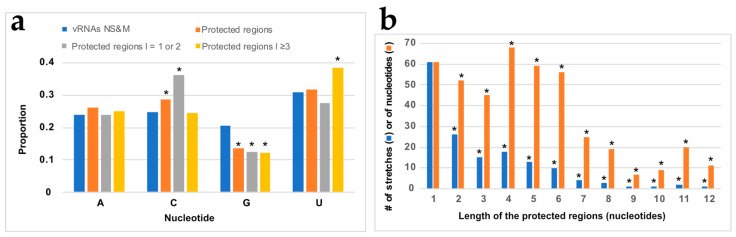
Base composition of the protected regions. (**a**) Base composition of all the protected regions (orange), the short protected regions (grey), and the long protected regions (yellow) compared to the composition of the NS and M vRNAs (dark blue). (**b**) Length distribution of the protected regions. Statistical tests of conformity were used to test whether the nt distribution in the protected regions differs from the nt distribution of the sequences of the NS and M vRNAs (**a**) or whether the observed distribution of the length of the protected regions differs from a random distribution of the protected nts along the vRNA sequences (**b**). *: *p* < 0.05.

Next, in order to determine if NP binding displayed some sequence preference, we analyzed the nt composition of the regions protected from NMIA modification upon the addition of NP (Figure 5a).

**Figure 6:**
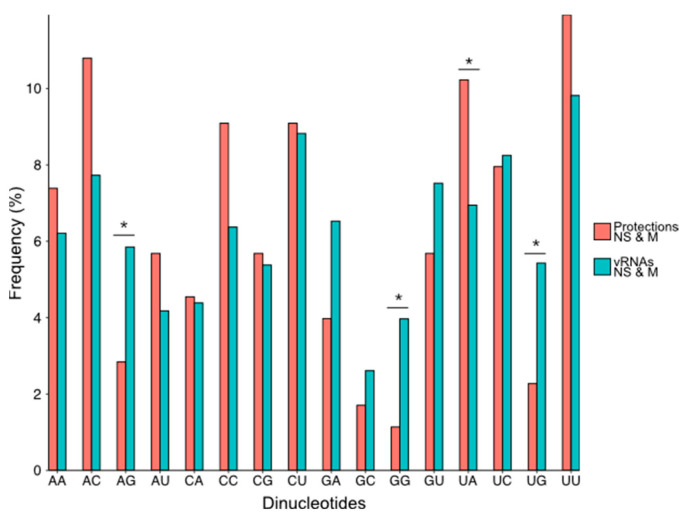
Frequency of the 16 dinucleotides in the NS and M vRNA sequences and in the regions of the vRNAs that are protected upon NP addition. Dinucleotides are written from 3′ to 5′ as per convention in the field of negative strand viruses. A statistical test of conformity was used to compare the distribution of the 16 dinucleotides in the protected regions and in the NS and M vRNA sequences. Since the number of each dinucleotide in the protected region was low, *p* values < 0.10 (*) were considered significant.

Compared to their frequency in the NS and M vRNA sequences, G residues are underrepresented by 1.5-fold in the regions that became protected upon the addition of NP, while the frequency of C residues is slightly but significantly over-represented in the protected regions closely matched the frequency of theses nts in NS and M vRNAs (Figure 5a). Since protected regions varied from one to twelve nts (Figure 5b and Data S2), we compared the base composition of the short protected regions (one or two nts in length) with the longer ones (three to twelve nts in length). This distinction reveals that C residues are significantly over-represented in short-protected regions, whereas U residues are significantly over-represented in the longer-protected regions (Figure 5a). This suggests that, except for a preference for non-G residues, the global base composition is not the only determinant of NP binding and prompted us to analyze the frequency of dinucleotides in the regions protected by NP compared to their frequency in the NS and M vRNAs (Figure 6).

As a general trend, dinucleotides containing at least one G residue were less frequent in the protected regions than in the whole of the NS and M vRNAs, and this difference was significant for three of them (AG, GG, UG) (Figure 6). On the contrary, the dinucleotide UA was significantly more frequent in the protected regions compared to the whole NS and M vRNA sequences (Figure 6). Quite surprisingly, the frequency of the CU and UC dinucleotides was similar in the protected regions and the NS and M vRNA sequences (Figure 6), even though C and U residues were over-represented in the short and long-protected regions, respectively (Figure 5a).

Considering a stoichiometry of ~1 NP per 24 nts [7,8], there should be ~37 and ~43 NP binding sites in the NS and M vRNAs, respectively. We observed 59 and 96 stretches of protected nucleotides in the NS and M vRNAs, respectively (Data S2), but several of these protected stretches lie in close proximity to each other in the vRNA secondary structures models (Figure 4a,b), suggesting that in some cases two or more protected stretches could correspond to a single NP binding site. For instance, in the NS vRNA, nts 195/218–219, 226/246–248/287, 257/273–274; 360–365/473, etc., might correspond to single NP binding sites (Figure 4a) and the same is true for nts 124–129/153–157; 203–205/208/211–212, 254–255/313, etc., in the M vRNA (Figure 4b). Furthermore, large junctions that connect several helices often display several protected nt stretches and are possibly protected by binding of a single NP protein: e.g., junctions 348–355, 397–407/437–439, 549–563/580–592, and 778–782/857–874 in the NS vRNA (Figure 4a), and junctions 201–218, 246–247/323–335, 355–357/462–469/490–491/1006, 365–370/394–398/428–439, 540–551/607–616/828–832, and 647–648/713/792–798, etc. in the M vRNA (Figure 4b). This suggests that, while protections can theoretically reflect either direct binding of NP or new base-pairings induced by NP binding, most protections likely correspond to NP footprints.

To test this hypothesis, we used the GUUGle v1.2 software [53] to identify sequences that could base-pair with the longest protected nt stretches. Of the eight protected sequences that are at least 8 nts in length, four protected regions have no partner sequence to form a stable helix. Amongst the four remaining protected regions, only one can potentially base-pair with a region that partially becomes protected upon the addition of the NP protein: nts 588–595 in the NS vRNA can potentially base-pair with nts 507–514, but the resulting helix would consist of 4 G-U and 4 A-U base-pairs and would therefore be fairly unstable. This reinforces the idea that a large fraction of the protections observed upon the addition of NP corresponds to NP footprints rather than the formation of new base pairs. Interestingly, the number of nts that became protected upon NP addition is similar to the number of nts that showed increased reactivity: in the NS and M vRNAs, respectively, 178 and 260 nts showed decreased reactivity, while 186 and 247 nts displayed increased reactivity upon NP addition (Figure 4). Since increased reactivity reflects the destabilization of helixes, whereas, as discussed above, most reduced reactivity does not reflect the formation of new base pairs, our results indicate that NP strongly destabilized the secondary structures of the NS and M vRNAs.

### 3.4. Using SHAPE Probing Data to Model the Secondary Structure of the vRNA/NP Complexes

**Figure 7:**
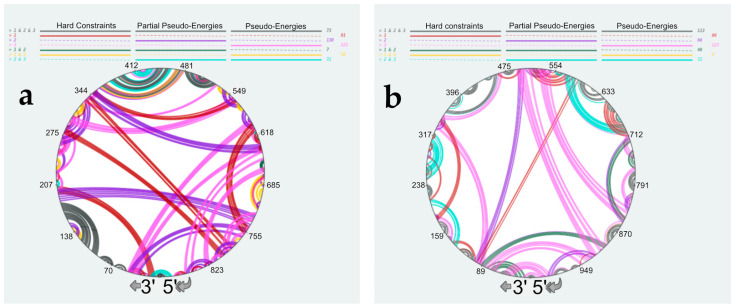
Comparison of the predicted secondary structures of the NS (**a**) and M (**b**) vRNAs complexed with NP. SHAPE data were treated as hard constraints, partial pseudo-energies, or pseudo-energies (See main text for detailed explanation). Structural elements that are common to three or two predicted structures or exist solely in one structure are color-coded as indicated at the top of the panels. Nucleotides of the vRNA are numbered from 3′ to 5′ as per convention in the field of negative strand viruses.

The common way to incorporate SHAPE probing data during secondary structure modeling is to implement them as pseudo-energies [48,49]. However, in the case of the vRNA/NP complexes, both base-pairing and NP binding can protect nts from modification by NMIA. Using the complete set of SHAPE reactivities as pseudo-energies to model the RNA secondary structure will likely favor the base-pairing of nts that are bound to NP, resulting in incorrect secondary structure models (Figures S4a and S5a). Nevertheless, SHAPE data contain useful information as nts with a SHAPE reactivity >0.8 are most likely unpaired. Therefore, we tested two different approaches to account for the high reactivity SHAPE values. In the first one, termed “partial pseudo-energies,” we only treated SHAPE values > 0.8 as pseudo-energies (Figures S4b and S5b) [36]. Alternatively, we used SHAPE values > 0.8 as hard constraints, forcing the corresponding nts to be unpaired in the secondary structure models (Figures S4c and S5c). Next, we compared the secondary structure models obtained using these three different approaches to take the SHAPE data into account using RNAStructViz [39] (Figure 7).

This analysis indicated that in 564, 482, or 454 out of the 890 nts, the NS vRNA are base-paired when the SHAPE data are treated as pseudo-energies, partial pseudo-energies, or hard constraints, respectively; for vRNA M, the corresponding figures are 614, 598, and 508, respectively. As expected, treating the high SHAPE reactivities as hard constraints resulted in the secondary structures with the lowest number of base pairs (Figures S4 and S5, and Figure 7). Figures S4 and S5, and Figure 7 reveal that the predicted secondary structures strongly depend on the way SHAPE data are used to constraint RNA modeling. Indeed, only 146 nucleotides of vRNA NS (Figure S4 and Figure 7a) and 226 nucleotides of vRNA M (Figure S5 and Figure 7b) form the same base-pairs in the secondary structure models generated by treating SHAPE reactivities as hard constraints, partial pseudo-energies, or pseudo-energies. Interestingly, these common base pairs correspond almost exclusively to local structures (Figure 7), with only one common helix in each vRNA involving nucleotides ~100 nucleotides apart: helix 362–364/472–474 in vRNA NS (Figure S4) and helix 359–364/455–460 in vRNA M (Figure S5). Of note, these local structures were also predicted to exist under the NoNP and ProtK conditions (compare Figure 2 with Figure 7), suggesting that they are particularly likely to exist. By contrast, different long-range interactions were predicted, depending on how the SHAPE data were used during RNA modeling (Figure 7a,b). Of note, using partial pseudo-energies instead of pseudo-energies did not reduce the number of highly reactive nts that were predicted to be base-paired (40 nts versus 30 nts for NS vRNA, and 30 nts versus 35 for M vRNA) (Figures S4a,b and S5a,b). This suggests that more information on NP binding sites is needed to predict the secondary structure of vRNA complexed with NP with high confidence.

## 4. Discussion

Initially, RNA chaperones were defined as proteins that transiently bind to RNA and remodel its structure [41,54]. However, many viral proteins that remain associated with the RNA to which they bind were found to affect the RNA structure when they were removed by protease degradation and/or phenol/chloroform extraction, and by extension, are also considered as RNA chaperones [31,32,33,42]. During IAV replication, the nascent vRNA and complementary RNA associate with NP as soon as they are synthesized, and NP is transiently displaced from the vRNA template during replication and transcription [2,55,56]. Despite tight NP binding at a mean ratio of one protein per 20 to 24 nts [7,8], stable and transient secondary structures present in vRNAs play crucial roles in packaging [6,9,21,57] and replication [58], respectively. Therefore, it is essential to understand to which extent NP transiently or permanently affects vRNA structure.

The vRNA structure is not visible in cryo-EM structures of authentic vRNPs [3,51,52], and while recent structures of NP/RNA complexes [20,59] help us understand how vRNPs are organized, they do not reveal how RNA structures can protrude from the central vRNP helical structure. Therefore, chemical probing [45,46] remains an important tool for the investigation of the impact of NP on the vRNA structure. Several recent studies used dimethyl sulfate and/or SHAPE reagents to decipher the vRNA structure in the context of vRNPs in viral particles or infected cells, as well as on deproteinated or in vitro transcribed vRNA [27,60,61]. One of these studies concluded that the NP protein has limited RNA chaperone activity [27], in agreement with our present study, which showed limited SHAPE reactivity differences between the NoNP and ProtK conditions (Table 2, Figures S1 and S2, and Figure 3). However, our detailed analysis of the secondary structure predicted under the NoNP and ProtK conditions suggests that the NP RNA chaperone activity, although limited, may be functionally relevant, as it may remodel long-range interactions that constrain the overall vRNA secondary structure (Figure 2 and Figure 3). For instance, the NP RNA chaperone activity may affect the formation of transient RNA structures during RNA replication [58,62].

In keeping with previous studies [27,61], we observed that the SHAPE reactivities of the vRNA/NP complexes strongly differ from those obtained under the NoNP and ProtK conditions (Table 3 and Table 4 and Figure 4). Protection against modification by NMIA could, in principle, reflect either new base pairs formed in the presence of NP or direct protection of the ribose-phosphate backbone by NP. Our analysis suggests that an important fraction of the longest protected regions does correspond to NP footprints. Indeed, up to 12 consecutive nts are protected upon NP addition (Figure 5b): this fits well with the fact that even though the positively charged groove of NP can accommodate six nts [12,15,16], up to ~12 nts are required for optimal binding [19] and the observation that IAV NP complexed with a 12-mer RNA can form long filaments resembling authentic vRNPs [14,20].

NP binds ssRNA with high affinity and little or no sequence specificity [14,18]. However, while most biophysical studies analyzed these interactions between NP and short ssRNAs [12,13,14,15], our study suggests that NP also binds to short bulges or internal loops and the adjacent helices (Figure 4). It would be interesting to test this hypothesis using short-model RNAs, especially as the currently available NP/RNA structures only show interactions of NP with ssRNA [20,59].

Our band-shift assay revealed a progressive shift of the vRNA/NP complexes when the protein concentration was progressively increased (Figure 1e), indicating that NP binds RNA with little or no cooperativity. Furthermore, our results show a slight but significant bias against G residues and towards C and U residues in the regions protected upon the addition of NP (Figure 5a and Figure 6). Accordingly, (UC)_6_ induces the formation of helical vRNP-like complexes, and this oligonucleotide has a higher affinity for NP than other dodecamers [20].

One of the main remaining challenges in deciphering the mechanisms underlying selective packaging of the eight IAV genomic segments and genetic reassortment is to gain detailed information about the secondary and three-dimensional vRNA structures within the context of vRNPs [5,21]. Previous IAV genome-wide chemical probing studies [27,60,61] together with the present work, bring important but indirect information about the vRNA structure in vRNPs and vRNA/NP complexes. In the absence of proteins, well-established methods allow the treatment of the reactivity of individual nts towards chemical probes as pseudo-energies in order to improve secondary structure predictions [36,48,49]. However, the presence of NP, which drastically affects the vRNA structure and directly protects RNA from modification (this work and references [27,61]), may preclude global chemical probing reactivities as pseudo-energies for RNA structure predictions. Since non-reactive nts may be either base-paired or protected by NP, we also predicted secondary structure using only the high SHAPE reactivities (>0.8) as “partial pseudo-energies” or hard constraints (Figure 7, Figures S4 and S5). Our comparison revealed that the secondary structure models obtained from these three approaches largely differ from one another, with only local secondary structures representing 1/4 to 1/3 of the total base pairs being found in the three predicted structures (Figure 7, Figures S4 and S5). This indicates that only a limited number of secondary structure elements existing in the vRNAs in the context of vRNPs or vRNA/NP complexes can be predicted with high confidence. Identifying the NP binding sites at single nt resolution would greatly help improve these predictions. There is no indication that the structural elements that can be predicted with high confidence are packaging signals. However, their conservation amongst the three predicted secondary structure models suggests they do not compete with alternate structures of similar energy, as expected for functional structures (except riboswitches).

## Figures and Tables

**Table 1:** Fluorescent primers used in the SHAPE experiments.

Primer Name	5′ Fluorophore	Sequence (5′→3′)
NS30V	Vic	GAT CCA AAC ACT GTG TCA AGC
NS30N	Ned	GAT CCA AAC ACT GTG TCA AGC
NS341V	Vic	CAT ACC CAA GCA GAA AGT GGC
NS341N	Ned	CAT ACC CAA GCA GAA AGT GGC
NS621V	Vic	CAG AGA TTC GCT TGG TGT TGC
NS621N	Ned	CAG AGA TTC GCT TGG TGT TGC
M20V	Vic	TGA AAG ATG AGT CTT CTA ACC
M20N	Ned	TGA AAG ATG AGT CTT CTA ACC
M312V	Vic	CAG TTA AAC TGT ATA GGA AGC
M312N	Ned	CAG TTA AAC TGT ATA GGA AGC
M622V	Vic	AGC AGA GGC CAT GGA TAT TGC
M633N	Ned	AGC AGA GGC CAT GGA TAT TGC
M843V	Vic	GAT CGT CTT TTT TTC AAA TGC
M843N	Ned	GAT CGT CTT TTT TTC AAA TGC

**Table 2:** Comparison of the SHAPE reactivity values of the nucleotides of the NS and M vRNA under the NoNP and ProK conditions.

NS vRNA (890 nts)		NoNP		
		Low	Intermediate	High
ProtK	Low	383	27	2
	Intermediate	59	45	7
	High	13	24	90
M vRNA (1027 nts)		NoNP		
		Low	Intermediate	High
ProtK	Low	413	54	2
	Intermediate	53	63	29
	High	12	38	100

Reactivity values were categorized as low (<0.4), intermediate, or high (>0.8). Negative and not determined values were excluded from the analysis.

**Table 3:** Comparison of the SHAPE reactivity values of the nucleotides of the NS and M of vRNAs under the NoNP and Comp conditions.

NS vRNA (890 nts)		NoNP		
		Low	Intermediate	High
Comp	Low	296	44	33
	Intermediate	84	25	34
	High	39	19	32
M vRNA (1027 nts)		NoNP		
		Low	Intermediate	High
Comp	Low	297	72	32
	Intermediate	106	45	45
	High	41	23	46

Reactivity values were categorized as low (<0.4), intermediate, or high (>0.8). Negative and not determined values were excluded from the analysis.

**Table 4:** Comparison of the SHAPE reactivity values of the nucleotides the NS and M of vRNAs under the ProtK and Comp conditions.

NS vRNA (890 nts)		ProtK		
		Low	Intermediate	High
Comp	Low	278	53	45
	Intermediate	69	29	40
	High	36	16	38
M vRNA (1027 nts)		ProtK		
		Low	Intermediate	High
Comp	Low	277	62	45
	Intermediate	114	42	46
	High	43	24	44

Reactivity values were categorized as low (<0.4), intermediate, or high (>0.8). Negative and not determined values were excluded from the analysis.

## Data Availability

All SHAPE reactivity data used in this article are tabulated in Supplementary Data S1.

## Supplementary Materials

The following supporting information can be downloaded at: https://www.mdpi.com/article/10.3390/v16030421/s1, Data S1: SHAPE reactivity values for the NS and M vRNAs under the NoNP, ProtK, and Comp conditions. Data S2: Regions of the NS and M vRNAs protected from NMIA modification upon the addition of NP protein. Table S1: Correlation of the SHAPE reactivity values obtained from three independent replicates for M and NS vRNAs under the NoNP, ProtK, and Comp conditions. Figure S1: Secondary structure models of the NS vRNA under the NoNP and ProtK conditions; Figure S2: Secondary structure models of the M vRNA under the NoNP and ProtK conditions; Figure S3: SHAPE reactivity profiles of the NS and M vRNAs; Figure S4: Predicted secondary structures of the NS vRNA complexed with the NP protein; Figure S5: Predicted secondary structures of the M vRNA complexed with the NP protein.
