# Supplementary material for: Structural Impact of the Interaction of the Influenza A Virus Nucleoprotein with Genomic RNA Segments
Source: Viruses. 2024 Mar 9;16(3):421. doi: 10.3390/v16030421 (PMC10974462; doi:10.3390/v16030421)
Supplement: Supplementary file 1 [file viruses-16-00421-s001.zip › Supplementary Material_revision.pdf]

Supplementary Material

Interaction of the influenza A virus nucleoprotein with genomic RNA segments: structural impact and implications for genome packaging

Erwan Quignon [1], Damien Ferhadian [1], Antoine Hache [1], Valérie Vivet-Boudou [1], Catherine Isel[1], Anne Printz-Schweigert [1+], Amélie Donchet[2], Thibaut Crépin [2], and Roland Marquet [1*]

[1] Université de Strasbourg, CNRS, Architecture et Réactivité de l'ARN, UPR9002, 67000 Strasbourg, France
[2] Institut de Biologie Structurale (IBS), CEA, CNRS, University Grenoble Alpes, Grenoble, France
+ This article is dedicated to the memory of Anne Printz-Schweigert
* Correspondence: r.marquet@ibmc-cnrs.unistra.fr; Tel.: +33 3 88 41 70 68

Supplementary Data

Data S1

See accompanying excel file

SHAPE reactivity values for the NS and M vRNA under the NoNP, ProtK and Comp conditions. Reactivity values of each replicate as well as the mean reactivity values are indicated. In these tables, numbering of the nucleotides is from 5′ to 3′of the vRNAs. The absence of data is indicated by -999.

Data S2

See accompanying excel file

Regions of the NS and M vRNAs protected from NMIA modification upon addition of NP protein. Sequence of the protected regions longer than one nucleotide are from 3′ to 5′.

Supplementary Table

See next page

Supplementary Figures

See next pages

Table S1: Correlation of the SHAPE reactivity values obtained from three independent replicates for M and NS vRNAs under the NoNP, ProtK and Comp conditions

	M vRNA				NS vRNA		
	NoNP	ProtK	Comp		NoNP	ProtK	Comp
Rep 1 vs rep 2	0,90	0,69	0,96	Rep 1 vs rep 2	0,79	0,83	0,96
Rep 1 vs rep 3	0,89	0,94	0,96	Rep 1 vs rep 3	0,74	0,84	0,96
Rep 2 vs rep 3	0,89	0,81	0,97	Rep 2 vs rep 3	0,76	0,89	0,95

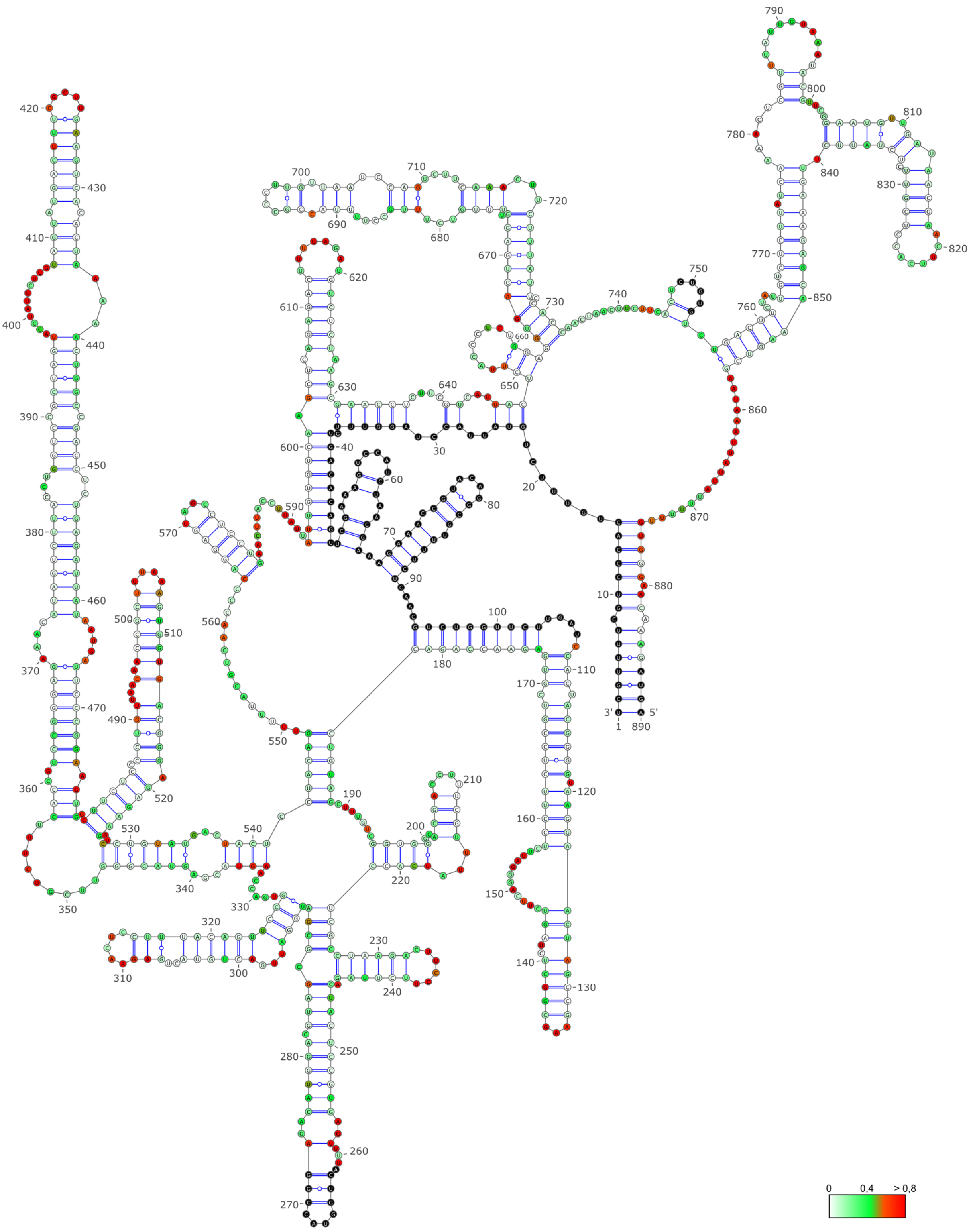

Figure S1a
(continued)

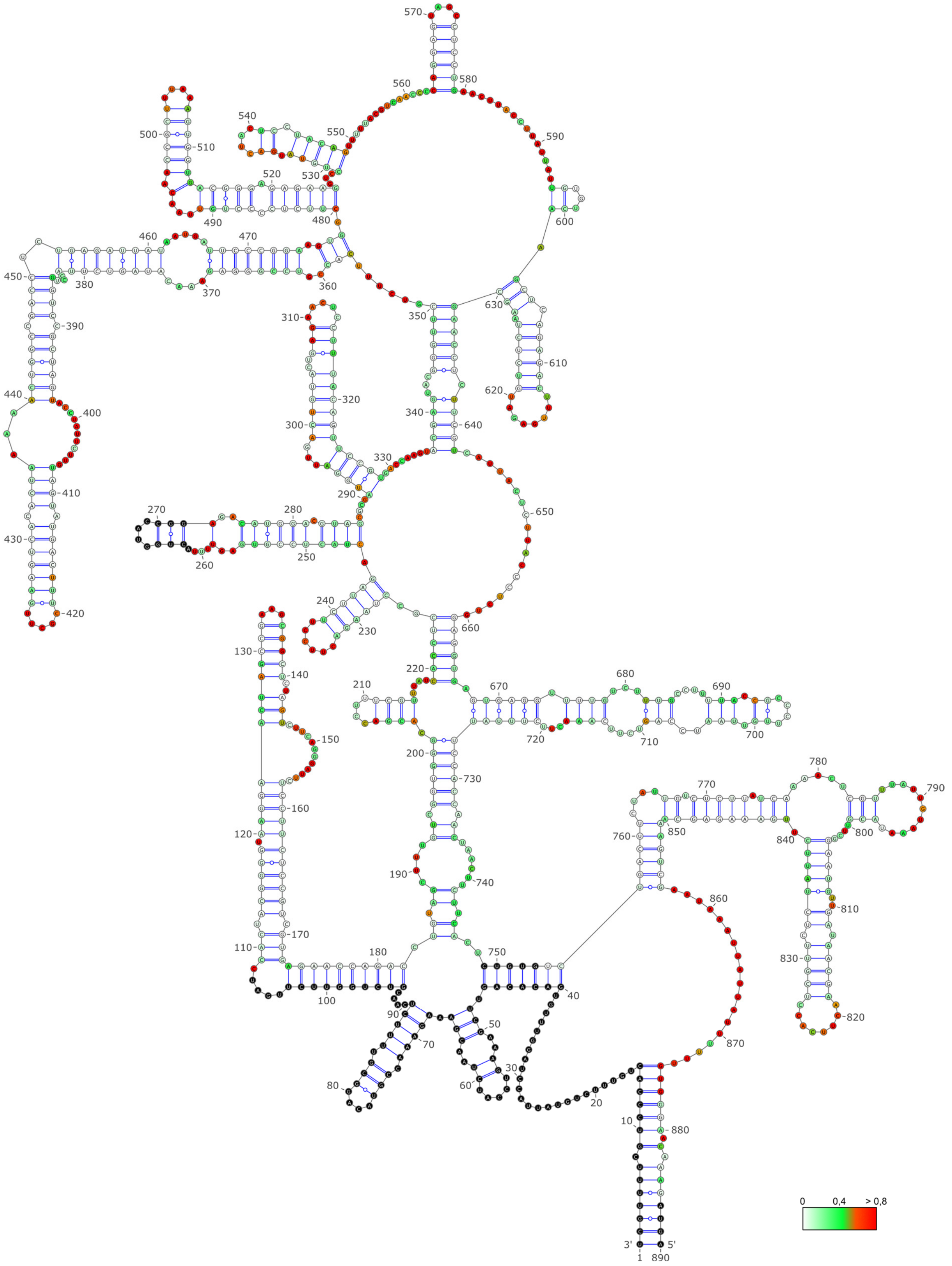

Figure S1b

Figure S1: Secondary structure models of the NS vRNA under the NoNP and ProtK conditions. These models were predicted using the SHAPE reactivity data obtained the under the **(a)** NoNP or **(b)** ProtK conditions as constraints. Nucleotides of the vRNA are numbered from 3′ to 5′ as per convention in the field of negative strand viruses. SHAPE reactivity values are color coded from white to red as shown in the insert. The absence of data is indicated by black circles.

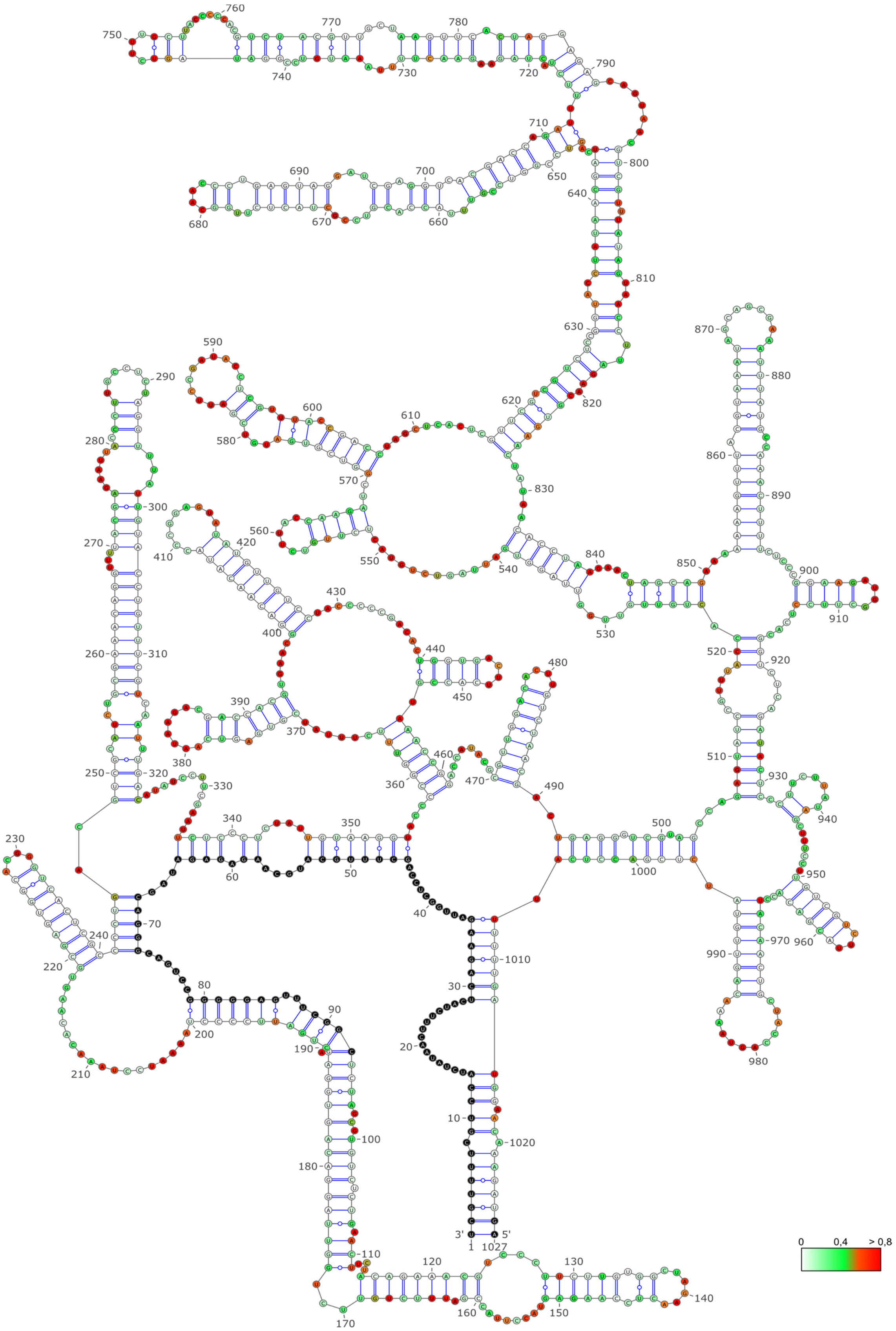

Figure S2a
(continued)

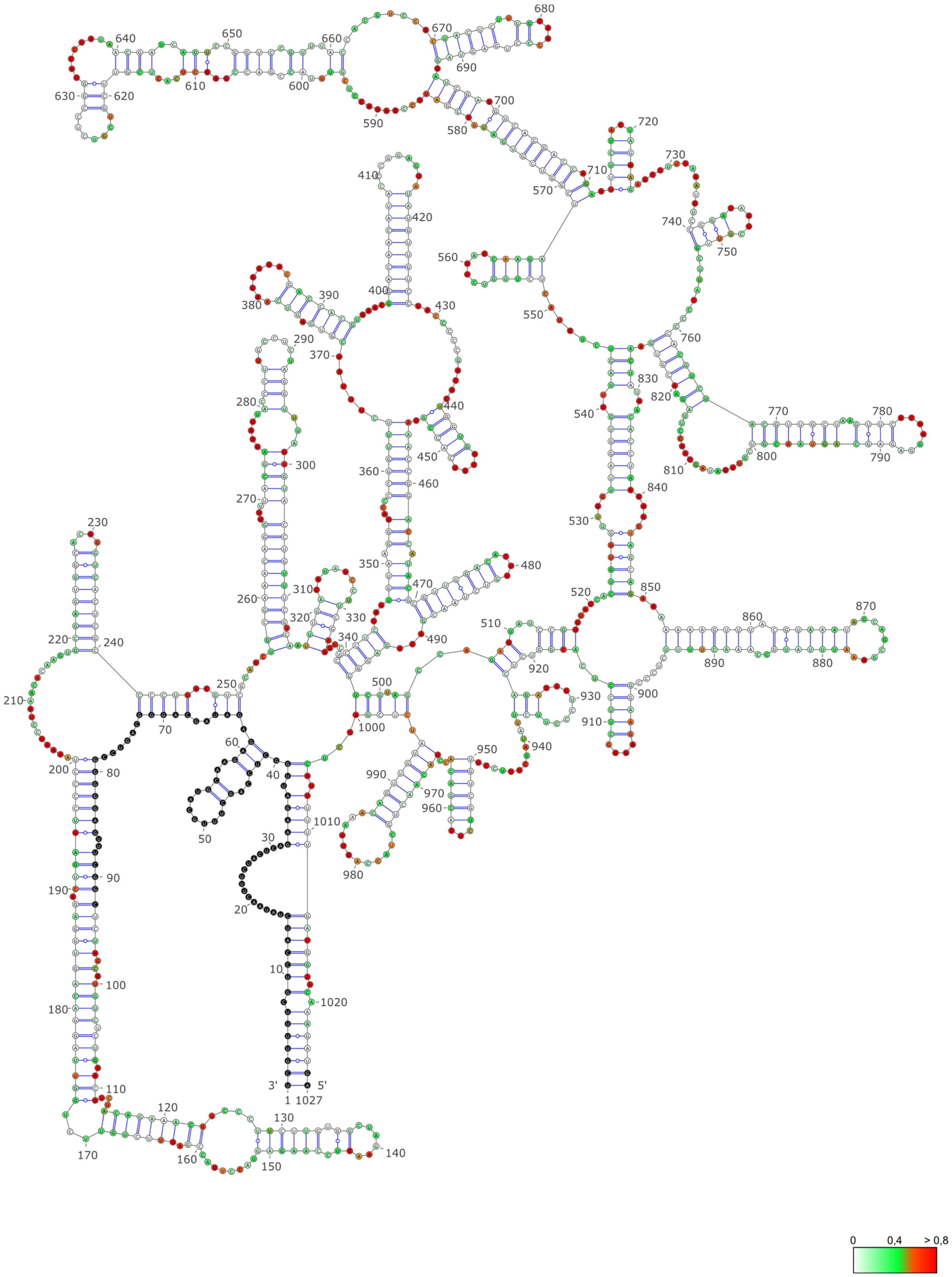

Figure S2b

Figure S2: Secondary structure models of the M vRNA under the NoNP and ProtK conditions. These models were predicted using the SHAPE reactivity data obtained the under the **(a)** NoNP or **(b)** ProtK conditions as constraints. Nucleotides of the vRNA are numbered from 3′ to 5′. SHAPE reactivity values are color coded from white to red as shown in the insert. The absence of data is indicated by black circles.

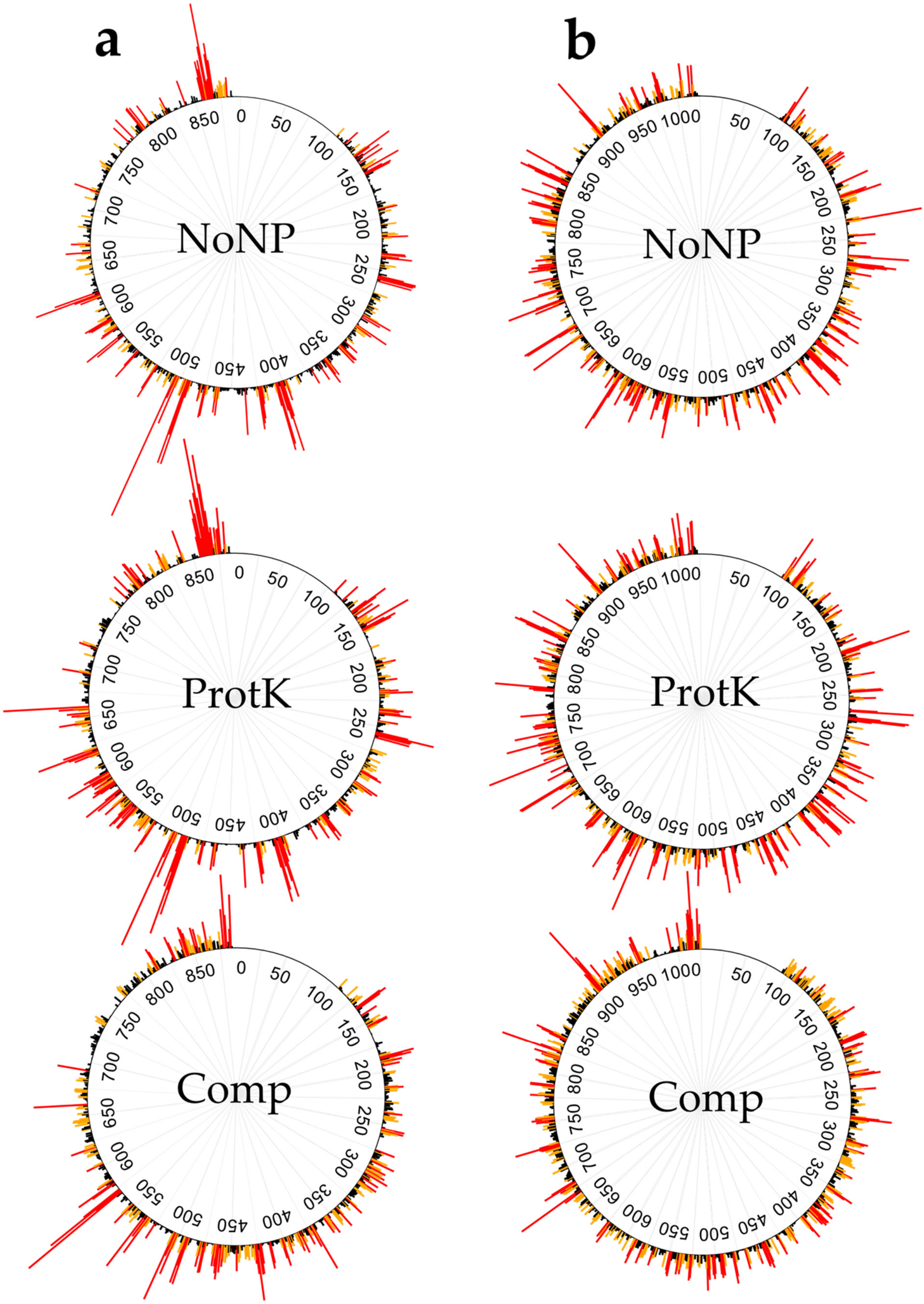

Figure S3: SHAPE reactivity profiles of the NS (a) and M (b) vRNAs. For each vRNA, the SHAPE reactivity profiles obtained under the NoNP (top), ProtK (middle) and Comp (bottom) are represented in a circular manner. Nucleotides of the vRNA are numbered from 3′ to 5′. Reactivity values are color-coded: <0.4 black, >0.8 red, intermediate: orange.

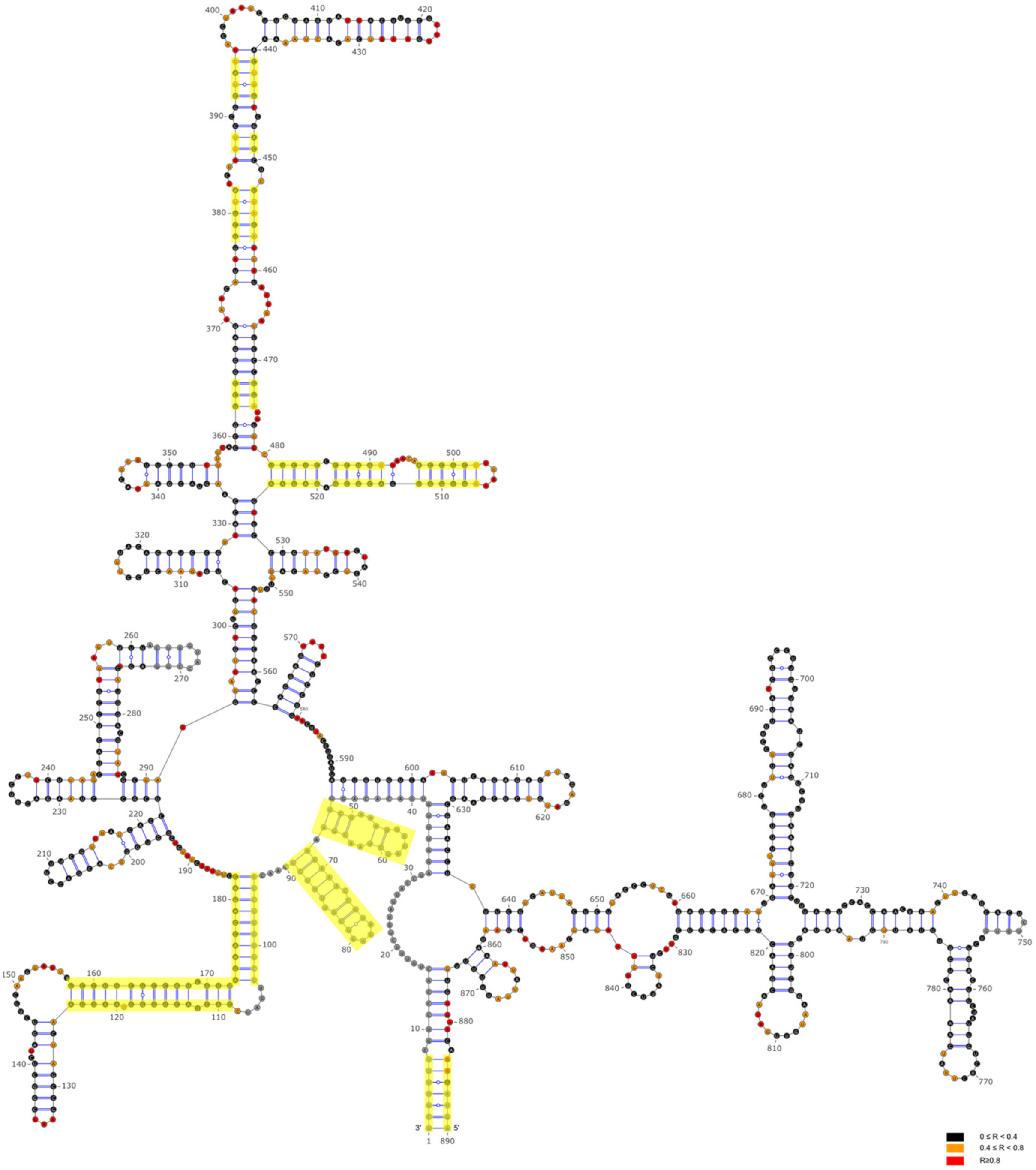
400
410
420
430
440
390
450
380
460
370
470
360
480
350
490
500
340
520
510
330
320
530
310
550
540
300
260
570
270
560
250
280
580
590
240
290
600
610
230
630
620
220
40
200
210
50
30
190
70
60
180
90
640
650
660
670
720
730
740
100
150
160
170
80
20
860
850
830
820
800
750
790
870
840
120
110
880
10
810
780
760
140
130
770
700
690
710
680
3'
5'
1
890
0 ≤ R < 0.4
0.4 ≤ R < 0.8
R≥0.8

Figure S4a

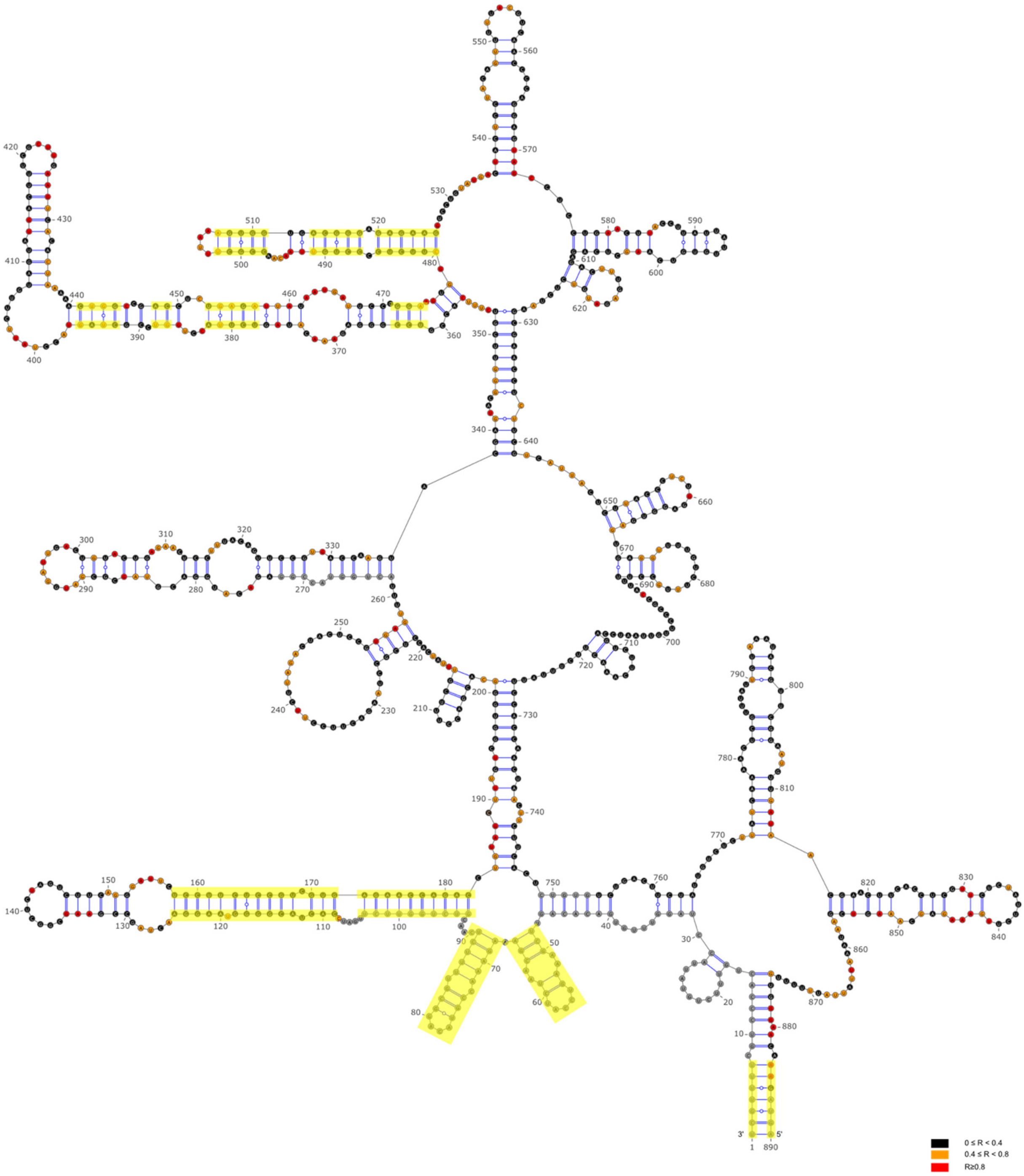

Figure S4b

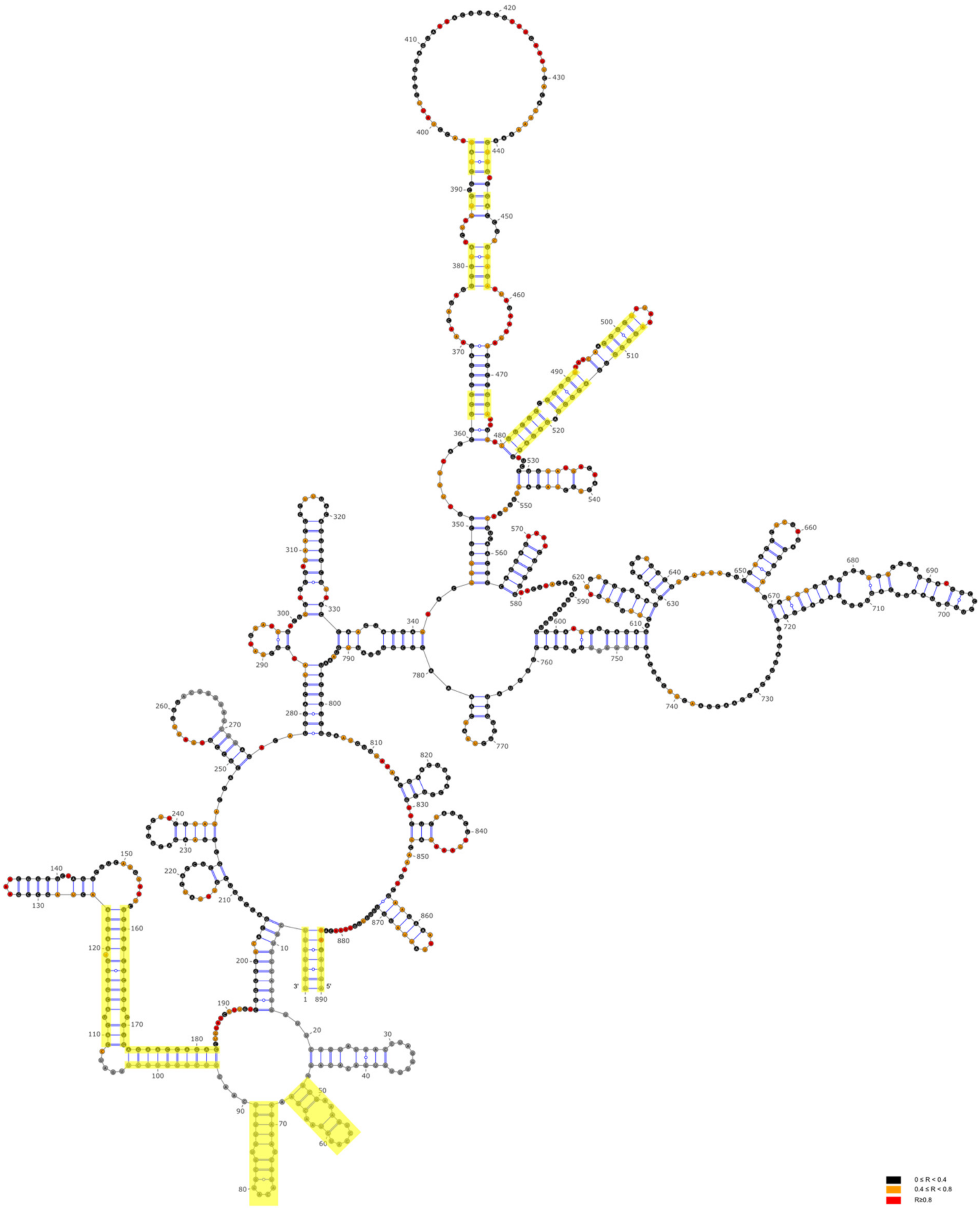

Figure S4c

Figure S4: Predicted secondary structures of the NS vRNA complexed with the NP protein. These models were predicted **(a)** using all SHAPE reactivities as pseudo-energies, **(b)** using only SHAPE reactivities > 0.8 as pseudo-energies or **(c)** using the SHAPE reactivities > 0.8 as hard constraints (see main text for more explanations). Reactivity of the nts in the vRNA/NP complex are color-coded as indicated in the inset. Base-pairs that are common to the 3 structures are highlighted in yellow.

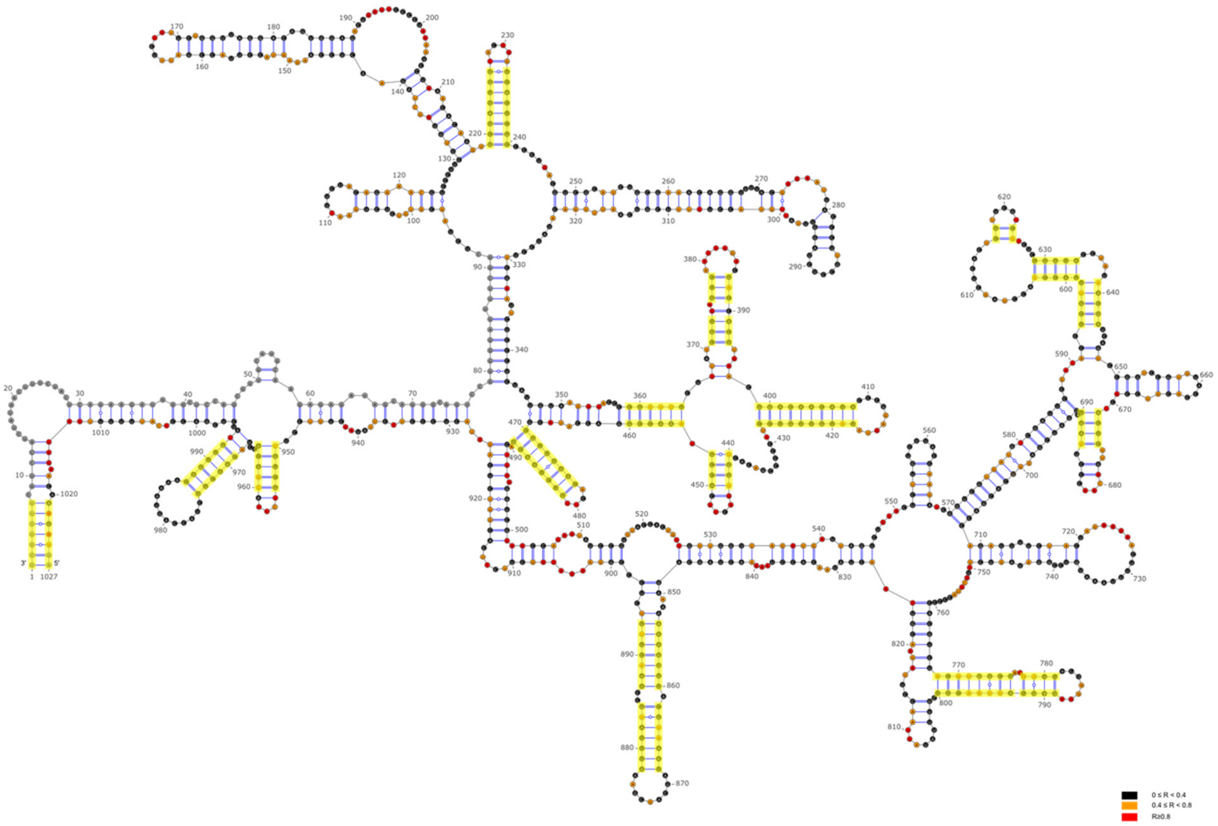

Figure S5a

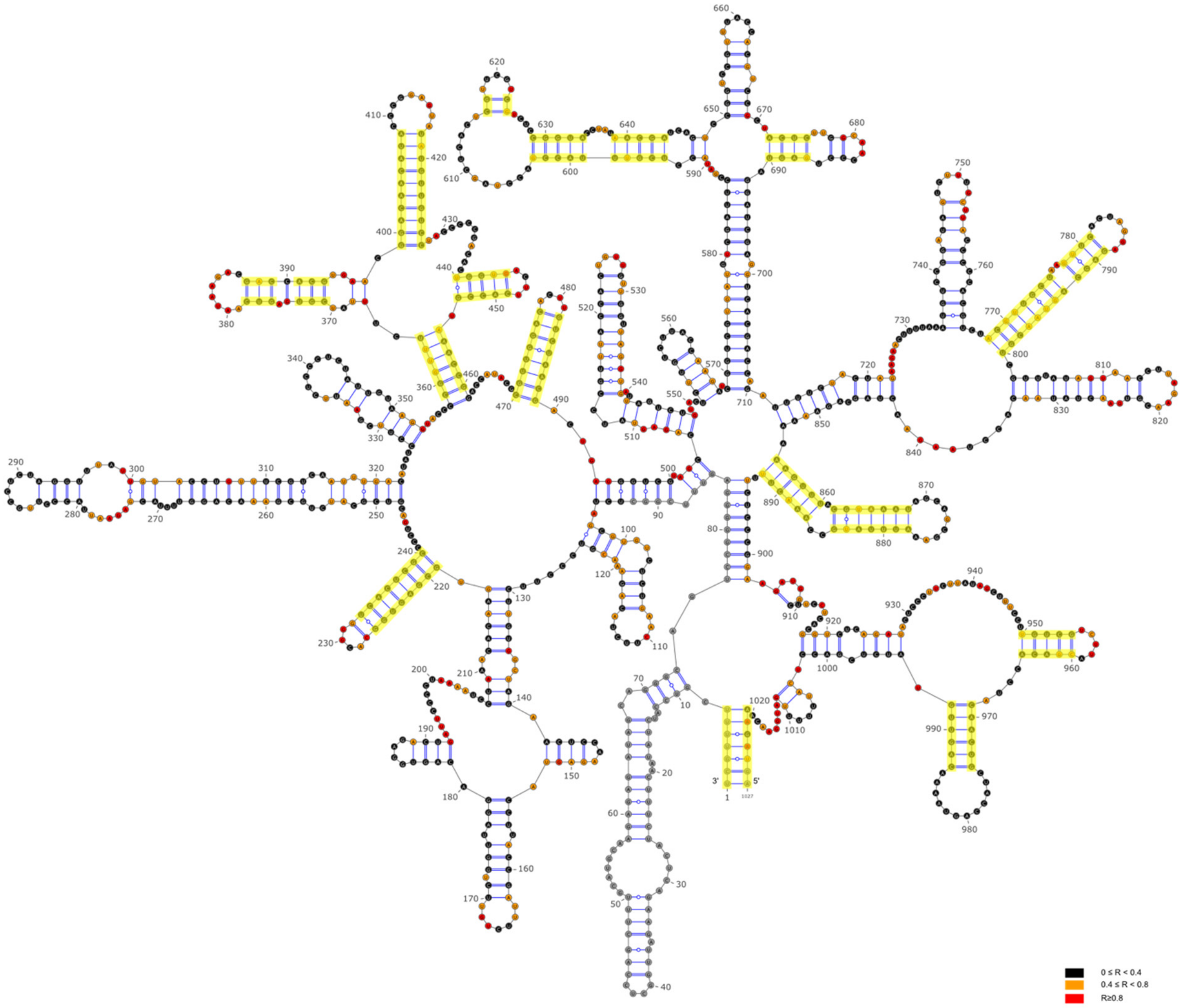

Figure S5b

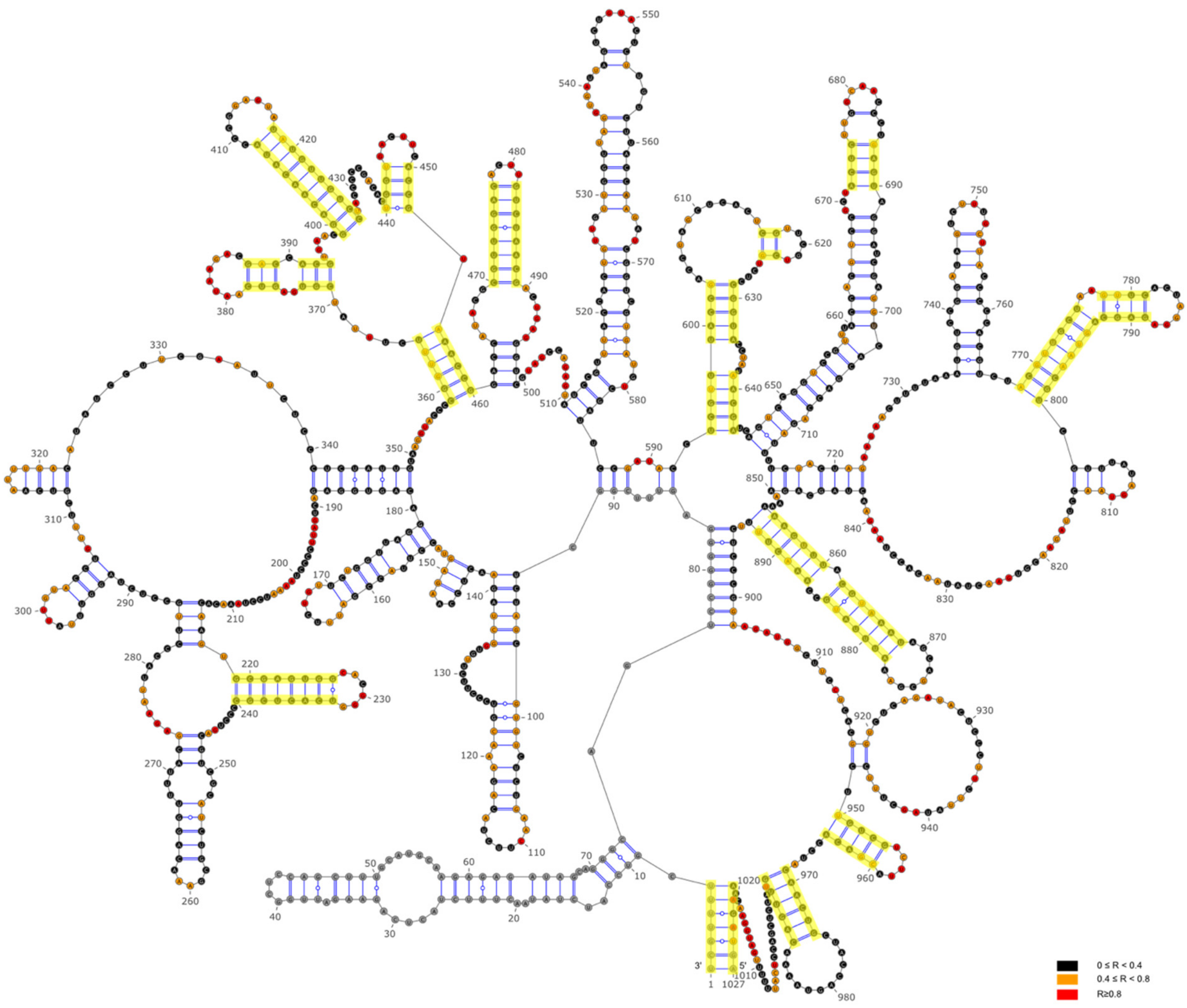

Figure S5c

Figure S5: Predicted secondary structures of the M vRNA complexed with the NP protein. These models were predicted **(a)** using all SHAPE reactivities as pseudo-energies, **(b)** using only SHAPE reactivities > 0.8 as pseudo-energies or **(c)** using the SHAPE reactivities > 0.8 as hard constraints (see main text for more explanations). Reactivity of the nts in the vRNA/NP complex are color-coded as indicated in the inset. Base-pairs that are common to the 3 structures are highlighted in yellow.
